# Phylogenetic Diversity of the *Bacillus pumilus* Group and the Marine Ecotype Revealed by Multilocus Sequence Analysis

**DOI:** 10.1371/journal.pone.0080097

**Published:** 2013-11-11

**Authors:** Yang Liu, Qiliang Lai, Chunming Dong, Fengqin Sun, Liping Wang, Guangyu Li, Zongze Shao

**Affiliations:** Key Laboratory of Marine Genetic Resources-State Key Laboratory Breeding Base, Third Institute of Oceanography of State Oceanic Administration, Fujian Provincial Key Laboratory of Marine Genetic Resources, Xiamen, China; Loyola University Medical Center, United States of America

## Abstract

Bacteria closely related to *Bacillus pumilus* cannot be distinguished from such other species as *B. safensis*, *B. stratosphericus*, *B. altitudinis* and *B. aerophilus* simply by 16S rRNA gene sequence. In this report, 76 marine strains were subjected to phylogenetic analysis based on 7 housekeeping genes to understand the phylogeny and biogeography in comparison with other origins. A phylogenetic tree based on the 7 housekeeping genes concatenated in the order of *gyrB*-*rpoB*-*pycA*-*pyrE*-*mutL*-*aroE*-*trpB* was constructed and compared with trees based on the single genes. All these trees exhibited a similar topology structure with small variations. Our 79 strains were divided into 6 groups from A to F; Group A was the largest and contained 49 strains close to *B. altitudinis*. Additional two large groups were presented by *B. safensis* and *B. pumilus* respectively. Among the housekeeping genes, *gyrB* and *pyrE* showed comparatively better resolution power and may serve as molecular markers to distinguish these closely related strains. Furthermore, a recombinant phylogenetic tree based on the *gyrB* gene and containing 73 terrestrial and our isolates was constructed to detect the relationship between marine and other sources. The tree clearly showed that the bacteria of marine origin were clustered together in all the large groups. In contrast, the cluster belonging to *B. safensis* was mainly composed of bacteria of terrestrial origin. Interestingly, nearly all the marine isolates were at the top of the tree, indicating the possibility of the recent divergence of this bacterial group in marine environments. We conclude that *B. altitudinis* bacteria are the most widely spread of the *B. pumilus* group in marine environments. In summary, this report provides the first evidence regarding the systematic evolution of this bacterial group, and knowledge of their phylogenetic diversity will help in the understanding of their ecological role and distribution in marine environments.

## Introduction

*Bacillus* is an important bacterial genus that consists of a heterogeneous group of aerobic or facultative anaerobic, endospore-forming, Gram-positive, rod-shaped organisms. Owing to their metabolic diversity and spore dispersal, *Bacillus* is ubiquitous in the environment. The genus *Bacillus* comprises 172 species recognized to date (http://www.bacterio.cict.fr/b/bacillus.html), most of which are from terrestrial environments. The strains in *Bacillus* are divided into the following 5 groups based on phylogenetic analysis of the 16S rRNA gene sequence: the *B. cereus*, *B. megaterium*, *B. subtilis*, *B. circulans* and *B. brevis* groups. Bacteria of *B. pumilus* belong to the *B. subtilis* group [1]. 

The bacteria of some *Bacillus* groups usually share high genetic homogeneity despite their phenotypic diversity, including the *B. cereus* group, with over 97 % 16S rRNA sequence similarity among *B. anthracis*, *B. cereus*, *B. weihenstephanensis*, *B. thuringiensis*, *B. mycoides*, *B. pseudomycoides*, *B. cytotoxicus*, *B. gaemokensis* and *B. manliponensis* [2]. However, the discrimination of these closely related bacteria has long been problematic. Many methods have been applied to identify and classify these *Bacillus* bacteria, including phenotypic characteristics, biochemical tests, fatty acid methyl ester (FAME) profiling [3], 16S rRNA gene sequencing [4,5], DNA fingerprinting [6], randomly amplified polymorphic DNA (RAPD) [7], restriction fragment length polymorphism (RFLP) [8], amplified fragment length polymorphism PCR (AFLP) [9] and multilocus enzyme electrophoresis (MLEE) typing. Recently, phylogenetic analyses based on single or multilocus sequence typing (MLST) of housekeeping genes, such as *rpoB* (RNA polymerase β subunit), *gyrB* (gyrase B subunit), 23S rRNA, *gyrA* and *pycA*, have been used frequently for this genus [10,11]. Indeed, these genes can effectively differentiate the strains of the *B. cereus* group and the *B. subtilis* group [12-15]. 

Due to the survivability of spores against harsh conditions, it remains unclear whether such spore-forming bacteria as *Bacillus* are indigenous to marine habitats. In fact, compared to their terrestrial relatives, little is known about the distribution and ecology of *Bacillus*, particularly in the deep sea [16,17]. According to biochemical tests, FAME profiling and partial 16S rRNA gene sequencing, *B. pumilus* was found to be the predominant species of cultivated *Bacillus* in the coastal environment of Cochin, India, followed by *B. cereus* and *B. sphaericus* [17,18].

In recent years, hundreds of *Bacillus* strains have been isolated in our lab from various marine environments of a wide geographic range, including deep sea, coastal and polar areas. We found that some *Bacillus* isolates closely related to *B. pumilus* are not easily distinguished from each other by 16S rRNA gene sequence alone. The *B. pumilus* group contains 5 species, *B. pumilus*, *B. safensis*, *B. stratosphericus*, *B. altitudinis* and *B. aerophilus*, which are nearly identical in 16S rRNA gene sequence, sharing similarity over 99.5%. In a phylogenetic tree of 16S rRNA gene sequences, this group is a neighbor of *B. atrophaeus* DSM 7264^T^, sharing similarity of less than 97.6%. Thus far, no systematic data are available to evaluate the diversity and evolution of this group. In an effort to understand the phylogeny, ecology and biogeography of this group, 76 marine strains and 3 type strains of this group were subjected to Multilocus Sequence Analysis (MLSA) based on 7 housekeeping genes and compared to 73 terrestrial isolates. 

## Materials and Methods

### Ethics statement

No specific permissions were required for collection of these the bacterial strains used in phylogenetic analysis in this study, as they are isolated from areas beyond national jurisdiction or from areas within the exclusive economic zone of China. Moreover, the sample sampling did not involve endangered or protected species. 

### Bacterial strains

A total of 76 strains of 5 species close to *B. pumilus* were chosen for the phylogeny study: 15 from the Pacific Ocean, 7 from the Indian Ocean, 3 from the Atlantic Ocean, 7 from the North Polar Region, 20 from the coast area of Fujian Province, 4 from the East China Sea and the Yellow Sea and 20 from the South China Sea (Table 1 and Figure S1 in File S1). These strains were deposited at Marine Culture Collection of China (MCCC).

**Table 1:** Bacterial isolates of the *B. pumilus* group strains used in MLSA analysis.

Strain No	Accession No^a^	Original No	Species^b^	Origin	Region	Elevation (m)	Types
1	1A00008	HYC-10	*Bacillus* sp.	Intestinal tract contents of fish	Xiamen island	0	B1
2	1A00112	HC21-A	*B. altitudinis*	Intestinal tract contents of fish	Xiamen island	0	A13
3	1A00242	Cr20	*B. altitudinis*	Sediment	Pacific Ocean	-5246	A1
4	1A00249	Cr30	*B. altitudinis*	Sediment	Pacific Ocean	-5246	A1
5	1A06451	FO-36b^T^	*B. safensis*	Clean-room air particulate	California	0	F7
6	1A00400	Mn48	*B. altitudinis*	Sediment	Pacific Ocean	-5000	A5
7	1A00401	Mn12	*B. altitudinis*	Sediment	Pacific Ocean	-5246	A1
8	1A00412	NHCd5-4	*B. altitudinis*	Sediment	South China Sea	-3649	A15
9	1A00420	02Co-3	*B. altitudinis*	Sediment	Pacific Ocean	-2869	A1
10	1A00439	Co21	*B. pumilus*	Sediment	Pacific Ocean	-5059	D3
11	1A00440	Co11	*B. altitudinis*	Sediment	Pacific Ocean	-5246	A4
12	1A00448	Ni27	*B. altitudinis*	Sediment	Pacific Ocean	-5059	A1
13	1A00466	Pb29	*B. altitudinis*	Sediment	Pacific Ocean	-5246	A2
14	1A00468	Pb71	*B. altitudinis*	Sediment	Pacific Ocean	-5059	A1
15	1A00482	Cr61	*B. altitudinis*	Sediment	Pacific Ocean	-5059	A1
16	1A01044	PA1A	*B. altitudinis*	Bottom water	Indian Ocean	-2488	A30
17	1A01364	8-C-1	*B. altitudinis*	surface water	Xiamen island	0	A22
18	1A01381	S70-5-12	*B. altitudinis*	Surface water	Indian Ocean	0	A20
19	1A02095	S2-5(2)2	*B. altitudinis*	Sediment	South China Sea	-15	A17
20	1A02227	2007/3/1	*B. altitudinis*	Sediment	Indian Ocean	-2434	A26
21	1A02467	DSD-PW4-OH8	*B. altitudinis*	Bottom water	South China Sea	-1762	A9
22	1A02468	mj01-PW1-OH23	*B. altitudinis*	Bottom water	South China Sea	-812	A23
23	1A02485	37-PW11-OH8	*B. altitudinis*	Bottom water	South China Sea	-1	A10
24	1A02775	IF1	*B. altitudinis*	Surface water	Yellow Sea	-30	A1
25	1A03121	A019	*B. altitudinis*	Surface water	East China Sea	0	A11
26	1A03126	A025	*B. altitudinis*	Surface water	Yellow Sea	-40	A31
27	1A04035	C16B11	*B. altitudinis*	Bottom water	Pacific Ocean	-1755	A1
28	1A04046	NH8D1	*B. altitudinis*	Sediment	South China Sea	-756	A35
29	1A04073	NH18E1	*B. altitudinis*	Sediment	South China Sea	-1550	A18
30	1A04526	NH21E_2	*B. safensis*	Sediment	South China Sea	-1184	F3
31	1A04568	NH21R_2	*B. altitudinis*	Sediment	South China Sea	-1184	A7
32	1A04638	NH24ET	*B. altitudinis*	Sediment	South China Sea	-1081	A16
33	1A05427	NH65B	*B. altitudinis*	Sediment	South China Sea	-1467	A12
34	1A05787	NH7I_1	*Bacillus* sp.	Sediment	South China Sea	-756	E1
35	1A05840	B204-B1-5	*B. safensis*	Sediment	South China Sea	-1467	F1
36	1A05860	BMJ03-B1-22	*B. safensis*	Sediment	South China Sea	-1100	F2
37	1A06638	CJWT7	*B. safensis*	Sediment	South China Sea	-11	F5
38	1A06692	HSGT11	*B. altitudinis*	Sediment	South China Sea	-11	A27
39	1A06774	HTZ_29	*B. altitudinis*	Sediment	South China Sea	-11	A19
40	1A06831	SCN16	*B. altitudinis*	Sediment	South China Sea	-11	A8
41	1A06858	SLN29	*B. safensis*	Sediment	South China Sea	-11	F4
42	1A06991	sxm20-2	*B. pumilus*	Sediment	Indian Ocean	-2089	D6
43	1A06996	B01-4	*B. pumilus*	Surface water	Pacific Ocean	0	D2
44	1A07053	B07-3	*B. pumilus*	Surface water	Pacific Ocean	0	D1
45	1A07134	BN04-13	*B. safensis*	Surface water	Pacific Ocean	0	F9
46	1A07286	P2-1B	*B. pumilus*	Sediment	Indian Ocean	-4735	D2
47	1A07375	S11-5	*B. altitudinis*	Sediment	Atlantic Ocean	-3217	A14
48	1A07587	C101	*B. altitudinis*	Sediment	Arctic Ocean	-4000	A32
49	1A07588	D21	*B. safensis*	Sediment	Arctic Ocean	-3566	F8
50	1A07590	D95	*B. safensis*	Sediment	Arctic Ocean	-2500	F8
51	1A07613	A1-1	*B. pumilus*	Sediment	Atlantic Ocean	-3310	D5
52	1A07638	A23-8	*B. altitudinis*	Sediment	Indian Ocean	-3879	A36
53	1A07644	A29-3	*B. pumilus*	Sediment	Indian Ocean	-2368	D4
54	1A01287	1A-5	*B. altitudinis*	Coral	Dongshan island	-2	A28
55	1A07606	2A-2	*B. altitudinis*	Coral	Dongshan island	-2	A33
56	1A07656	P1C-6	*B. altitudinis*	Coral	Dongshan island	-2	A25
57	1A07600	P3A-7	*B. altitudinis*	Coral	Dongshan island	-2	A34
58	1A05459	P6A-8	*B. altitudinis*	Coral	Dongshan island	-2	A28
59	1A05490	J33-1	*B. pumilus*	Sediment	Yellow Sea	-31.5	D4
60	1A00023	HYg-9	*B. safensis*	Intestinal tract contents of fish	Xiamen island	0	F8
61	1A00118	HYG-22	*B. altitudinis*	Intestinal tract contents of fish	Xiamen island	0	A24
62	1A07052	NP-4	*B. safensis*	Surface water	Arctic Ocean	0	F8
63	1A06453	DSM 27^T^	*B. pumilus*	Soil	/	/	D7
4	1A08385	15-B04 10-15-3	*B. safensis*	Sediment	Bering Sea	-3873	F6
5	1A08208	R06B32	*Bacillus* sp.	Sediment	Arctic Ocean	-44.5	C1
66	1A08151	C2-2	*B. pumilus*	Sediment	Atlantic Ocean	-3452	D2
67	1A08152	DW2J2	*B. pumilus*	White shrimp	Shrimp farm	0	D1
68	1A08153	DW3XJ7	*B. pumilus*	White shrimp	Shrimp farm	0	D1
69	1A08154	XW1-6	*B. pumilus*	Aquaculture water	Shrimp farm	0	D1
70	1A08372	DW5-4	*Bacillus* sp.	Aquaculture water	Shrimp farm	0	C1
71	1A08155	DW3-7	*B. safensis*	Aquaculture water	Shrimp farm	0	F8
72	1A08373	BS1	*B. altitudinis*	Bottom water	South China Sea	-1762	A1
73	1A00009	HYC-12	*B. altitudinis*	Intestinal tract contents of fish	Xiamen island	0	A37
74	1A08369	C70	*B. altitudinis*	Sediment	Arctic Ocean	-2790	A1
75	1A08156	DW2-3	*B. altitudinis*	Aquaculture water	Shrimp farm	0	A1
76	1A08157	DW3XJ1	*B. altitudinis*	White shrimp	Shrimp farm	0	A1
77	1A08370	DW2-4	*B. altitudinis*	White shrimp	Shrimp farm	0	A32
78	1A08371	XW3XJ7	*B. altitudinis*	White shrimp	Shrimp farm	0	A6
79	1A06452	41KF2b^T^	*B. altitudinis*	High-elevation air sample	Hyderabad/India	41000	A21

a The deposit accession No in MCCC (Marine Culture Collection of China).

b The name of these isolates were modified after phylogenetic analysis.

T Three type strains were marked.

/ The detailed information of *B. pumilus* DSM 27^T^ can not be found from reference.

In addition, 3 type strains, *B. pumilus* DSM 27^T^ isolated from soil [19], *B. safensis* FO-36b^T^ isolated by the Jet Propulsion Laboratory spacecraft-assembly facility of California in USA [20] and *B. altitudinis* 41KF2b^T^ isolated from air samples of high elevations (41,000 m) in India [21], were also included in the phylogeny study; these strains were purchased from DSMZ (Deutsche Sammlung von Mikroorganismen und Zellkulturen GmbH) in Germany. Unfortunately, 2 other type strains, *B. stratosphericus* and *B. aerophilus*, isolated from the same sample as *B. altitudinis* 41KF2b^T^, are no longer available in public collections or from the authors and therefore not included in the our analyses. The *gyrB* sequences of 73 strains were acquired from the NCBI database, and their detailed information is listed in Table S1 in File S1.

### DNA extraction

The strains were reactivated on a modified solid Luria-Bertani medium (10 g peptone, 5 g yeast extract, 10 g NaCl, 15 g agar and 1 L double-distilled water, pH 7.5) [22] and incubated at 37°C for 24 h. A suitable amount of cells on the plates were selected and transferred to 1.5 mL centrifuge tubes using sterile pipette tips. Genomic DNA was extracted using the SBS extraction kit (SBS Genetech Co., Ltd. in Shanghai, China) according to the manufacturer's instructions.

### PCR amplification and sequencing of 16S rRNA and housekeeping genes

The 16S rRNA gene was amplified by PCR using universal primers 27F and 1492R, and seven housekeeping genes were amplified using specific primers designed using Primer 5.0 (Table S2 in File S1). The genes were amplified under nearly the same conditions. In brief, each PCR mixture contained 1 µL genomic DNA, 1.25 U Ex Taq^TM^ DNA polymerase (TaKaRa), 4 µL dNTP mixture (2.5 mM of each dNTP), 1 µL each primer (20 µM), 5 µL 10×Ex Taq buffer (Mg^2+^ Plus) and sterile deionized water to a total volume of 50 µL. PCR was performed using a My Gen^TM^ L Series Peltier Thermal Cycle (Hangzhou Long Gene Scientific Instruments Co., Ltd, China). Each PCR product was separated by electrophoresis on a 1% agarose gel. The target PCR products were purified with the AxyPrep^TM^ PCR Clean up kit (Axygen Scientific, Inc., USA) according to the manufacturer's instructions and sequenced using the ABI3730xl platform (BGI Co., Ltd, China). 

The assembly and modification of the DNA sequences, including the 16S rRNA gene and seven housekeeping genes, were performed using DNAMAN 5.0 software. All sequences were deposited into the GenBank database; the accession numbers were listed in Table S3 in File S1.

### Phylogenetic analysis based on single gene analysis and MLSA

The determined sequences of the 16S rRNA gene and seven housekeeping genes were analyzed against sequences in the NCBI database using Blastn [23]. A substitution saturation assessment was performed for each gene sequence using DAMBE [24]. Recombination events in the DNA sequence alignments were evaluated using RDP 3.0 [25]. The genetic distances and sequence similarities of gene(s) were calculated using Kimura’s 2-parameter model [26] with the MEGA 5.0 software. The selective pressure on housekeeping gene was evaluated with the calculation of nonsynonymous (Ka) and synonymous (Ks) substitution rates （Ka/Ks）by the DnaSP 5.0 software [27].

The phylogenetic trees were constructed using the neighbor-joining (NJ) algorithm [28] with MEGA 5.0 [29]. The strengths of the internal branches of the resulting trees were statistically evaluated by bootstrap analysis with 1000 bootstrap replications. *B. cereus* ATCC 14579^T^ (GenBank accession: AE016877) was used as the outgroup. 

## Results

### Phylogenetic diversity revealed by 16S rRNA gene analysis

All the tested bacteria were subjected to a 16S rRNA gene analysis, even though they had been characterized (approximately 600 bp) prior to deposition in MCCC. Nearly the full-length 16S rRNA gene sequences (approximately 1513 bp) were obtained to further assess the taxonomic affiliation and phylogeny of the strains. 

The results demonstrated that the genetic distance of the 16S rRNA gene ranged from 0-0.005 (mean 0.002). Moreover, the number of alleles and polymorphic sites were only 7 and 10, respectively, and the proportion of polymorphic sites was 0.7%. In addition, the intraspecies similarities of 16S rRNA gene ranged from 99.6% to 100%, while the interspecies similarities were 99.5%-100%. These features of the 16S rRNA gene were presented in Table 2 and Table S4 in File S1. The 16S rRNA genes of the strains were highly conserved, and their similarities had overlap in intraspecies and interspecies, therefore it was unsuitable for the differentiation of these closely related strains. 

**Table 2:** Characteristics of the 16S rRNA gene, housekeeping genes and concatenated genes from 79 strains.

Locus	Length (bp)	Alleles No	Polymorphic site No /Percentage (%)	Mean G+C content (mol%)	K2P distance range	K2P distance mean
16S rDNA	1513	7	10/0.70	55.03	0.000-0.005	0.002
*gyrB*	717	35	170/23.71	42.20	0.000-0.110	0.053
*rpoB*	927	34	94/10.14	45.74	0.000-0.045	0.021
*aroE*	900	31	253/28.11	42.20	0.000-0.159	0.080
*mutL*	828	38	202/24.40	45.30	0.000-0.135	0.068
*pycA*	864	37	197/22.80	42.93	0.000-0.128	0.065
*pyrE*	546	33	160/29.30	46.51	0.000-0.179	0.085
*trpB*	867	29	232/26.76	44.77	0.000-0.149	0.072
MLSA	5649	54	1308/23.00	44.20	0.000-0.110	0.053

**Figure 1:**
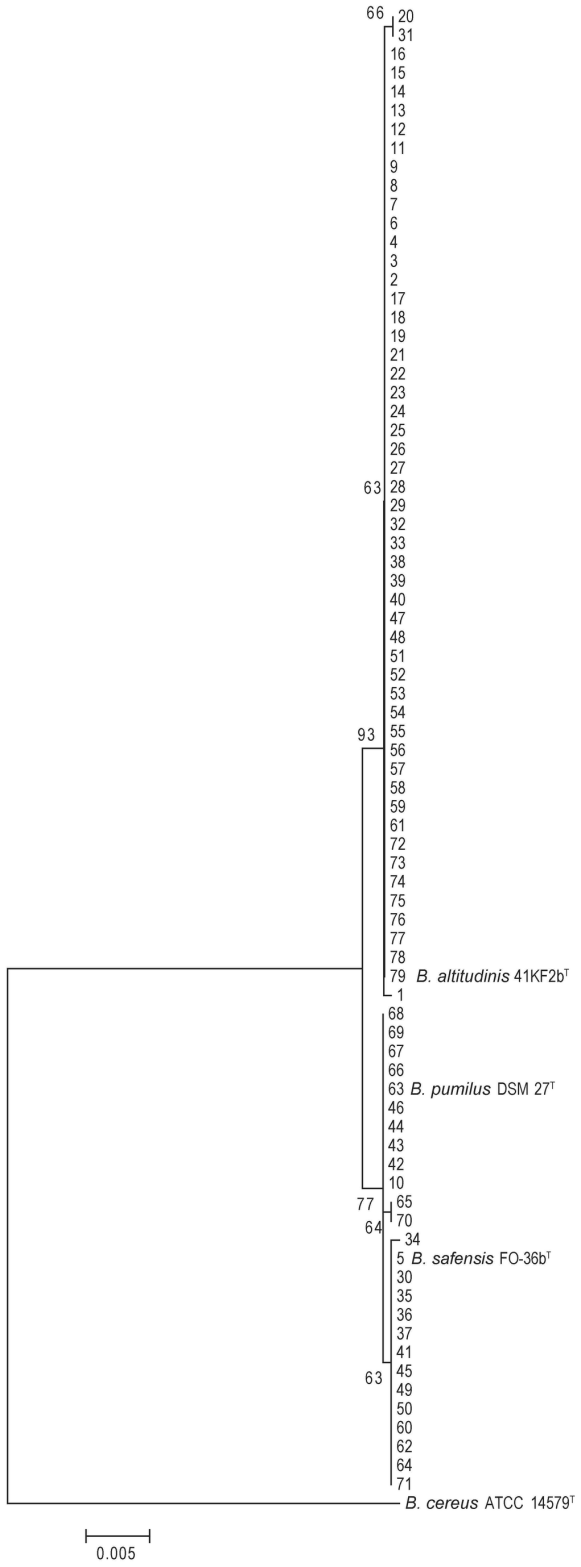
Phylogenetic tree based on the 16S rRNA genes of marine bacteria belonging to the *B*. *pumilus* group. The tree was constructed using the neighbor-joining method with MEGA 5.0. Bootstrap values over 50% (1000 replications) were shown at each node. Bar, % estimated substitution. *B*. *cereus* ATCC 14579^T^ was used as the outgroup.

Despite the high similarity, the phylogenetic tree of the 16S rRNA gene showed that the 79 strains were divided into two groups (Figure 1). The large group contained our 52 isolates, which were close to the type strain *B. altitudinis*; the small group contained 24 strains that we isolated, which were close to the type strains *B. pumilus* and *B. safensis* and cannot be distinguished by their 16S rRNA gene.

### Characteristics of seven housekeeping genes

To discriminate among the closely related bacteria, the housekeeping genes *gyrB*, *rpoB*, *aroE*, *mutL*, *pycA*, *pyrE* and *trpB* were chosen for analysis; 79 strains, including the 3 type strains, were analyzed. The characteristics of each housekeeping gene, such as the gene length, number of alleles, polymorphic sites, the mean G+C content, the genetic distance and the similarity range were shown in Table 2 and Table S4 in File S1.

The correlation of genetic distance between two housekeeping genes was calculated (Table S5 in File S1). An analysis of the characteristics and genetic distance of the housekeeping genes (Table 2) demonstrated that all the housekeeping genes showed remarkably higher resolution than the 16S rRNA gene (Table 2). Among the 7 housekeeping genes, the *pyrE* gene exhibited the highest resolution, with 29.3% polymorphic sites and the largest genetic distance range (0-0.179), whereas *rpoB* exhibited the lowest resolution. Although *mutL* had a higher allele number (38) than other genes, its polymorphic site percentage was less than many others. Specifically, *gyrB* displayed a better differentiation among strains close to *B. altitudinis*; in contrast, *aroE* was more powerful for strains of *B. pumilus*, and *pyrE* was better for strains of *B. safensis* (Table S6 in File S1).

**Figure 2:**
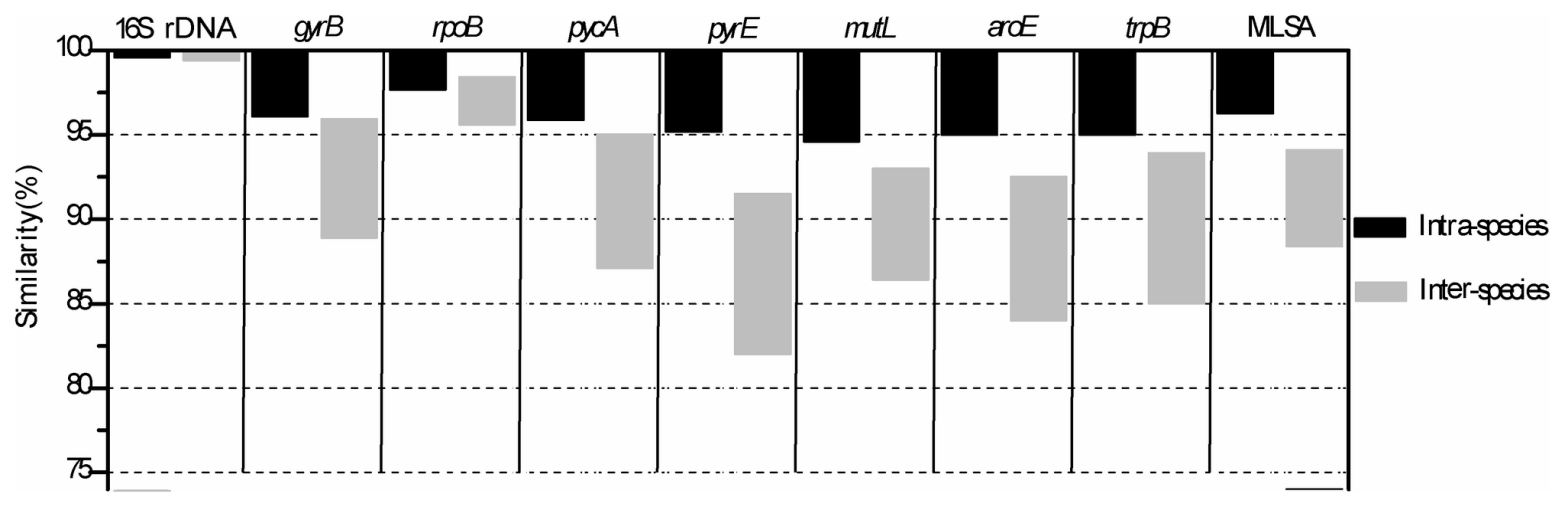
Intraspecies and interspecies similarity ranges of housekeeping genes in the *B. pumilus* group.

Further, DNA sequence similarity ranges of the 7 genes at intraspecies and interspecies levels were analyzed with the MEGA 5.0 software. These 79 strains were divided into 6 species, three of which were established species and others were potential novel species as documented below. The similarity ranges at intraspecies and interspecies levels were shown in Table S4 in File S1. An obvious gap between intraspecies and interspecies similarity ranges was observed in most housekeeping genes with exceptions of 16S rDNA and *rpoB* (Figure 2, Table S4 in File S1). For more details, the numbers of strain pairs within different similarity grades of the housekeeping genes of the 79 strains were shown in Table S7 and Figure S2 in File S1. These data indicates that 16S rDNA and *rpoB* were inappropriate for species discrimination among the bacteria of this group, while other genes showed a general interspecies similarity gap of 92% to 96%, and can serve in species discrimination, especially *pyrE* (92%-95%) and *aroE* (93-95%) Table S7 and Figure S2 in File S1), 

In addition, the Ka/Ks ratio of each housekeeping gene of different species and all the 79 strains was calculated, the results were displayed in Table S8 and Figure S3 in File S1. All the genes exhibited low Ka/Ks ratios ranging from 0.0000-0.1200 (Table S8 and Figure S3 in File S1), suggesting that they are under negative selection pressure. However, the ratios of Ka/Ks of each gene in different species were significant differences. The *pyrE* gene had the highest Ka/Ks ratio (0.1200) in *B. altitudinis*, while the gene *aroE* in *B. pumilus* and *B. safensis* were the highest, respectively 0.0800 and 0.0561. Even at interspecies level based on all the 79 strains, *pyrE* had the highest Ka/Ks ratio (0.0731). In contrast, *rpoB* had the lowest the Ka/Ks ratio, and was the most conserved among the seven housekeeping genes. 

### Phylogenetic diversity revealed by individual housekeeping genes

Prior to the phylogenetic analysis, these housekeeping genes were subjected to an examination of sequence substitution saturation and recombination events (data not shown). The saturation test of each housekeeping gene with DAMBE showed no sign of substitution saturation, and no recombination events were found in any of their tested housekeeping genes, as determined by program RDP-3.0. These results indicated that these sequences provided essential phylogenetic information.

Phylogenetic analyses based on each of the housekeeping genes were able to distinguish the strains at the species level. Moreover, the phylogenetic trees possessed nearly congruent topology structure (Figure S4-S10 in File S1). Specifically, the 79 strains were divided into 6 groups from A to F. Group A is the largest, containing 49 strains close to *B. altitudinis*; Group F is the second largest group, containing 13 strains belonging to *B. safensis*. Group D consisted of 13 strains attributed to *B. pumilus*. Additional three minor groups were revealed, Groups B, C and E, supported by only 1 to 2 strains each. These minorities represent putative novel taxa. 

Slight differences were also observed in some groups among the topologies of the seven trees. For example, *B. pumilus* was close to *B. altitudinis* in the phylogenetic tree of *gyrB*; in contrast, *B. pumilus* is close to *B. safensis* in other trees. In addition, the position of Groups B, C and D varied in the trees of *gyrB*, *rpoB* and *mutL*. For instance, Group B was closer to Group A in the phylogenetic trees of *gyrB*, *aroE*, *mutL*, *pyrE* and *trpB*, whereas Group B was closer to the groups in the large cluster of *B. safensis* and *B. pumilus* in the tree of *pycA*. Other small differences were also observed in the trees, as shown in the supplementary materials (Figure S4-S10 in File S1).

### Phylogeny based on the concatenated housekeeping genes

**Figure 3:**
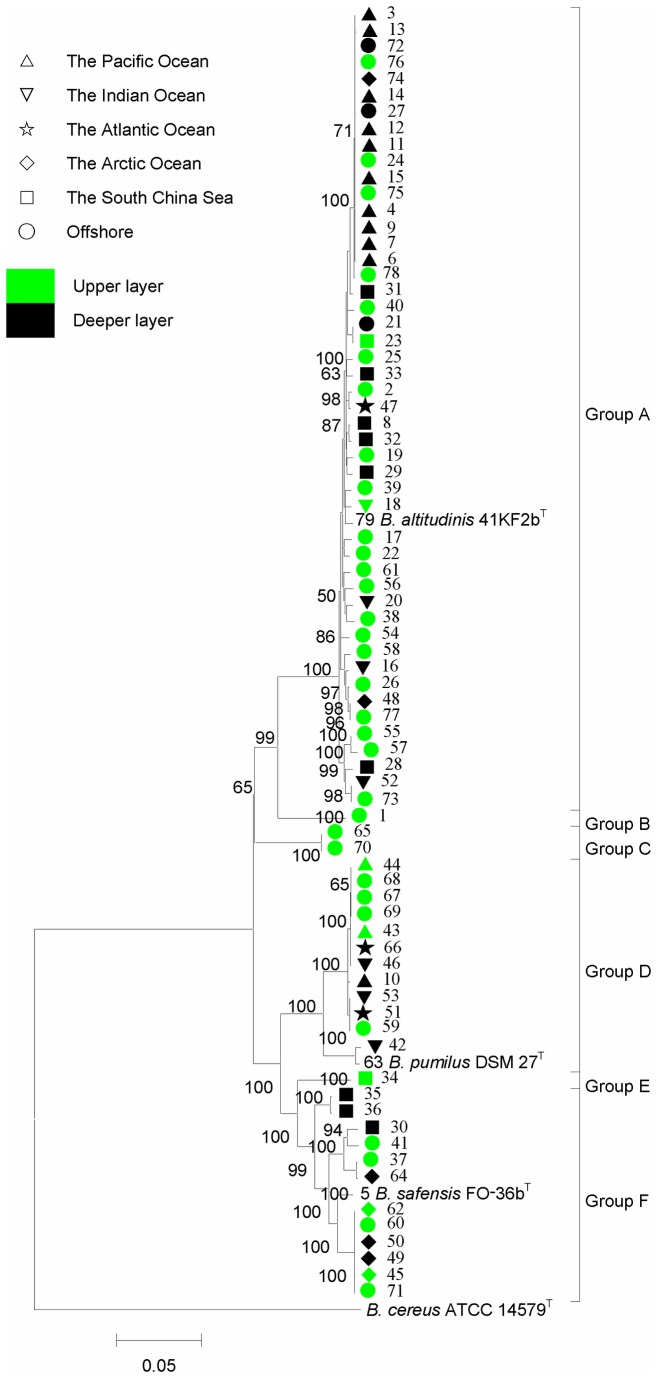
Phylogenetic tree based on seven housekeeping genes concatenated of marine isolates of the *B*. *pumilus* group. The tree was constructed using the neighbor-joining method with MEGA 5.0. Bootstrap values over 50% (1000 replications) were shown at each node. Bar, % estimated substitution. *B*. *cereus* ATCC 14579^T^ was used as the outgroup.

The seven housekeeping genes were concatenated in the order of *gyrB*-*rpoB*-*pycA*-*pyrE*-*mutL*-*aroE*-*trpB* (5649 bp) to reexamine the phylogeny of the 79 strains (Figure 3). The new phylogenetic tree showed a similar topology as the trees described above based on a single gene but was more elaborate and stable. 

Specifically, Group A consisted of 49 strains belonging to *B. altitudinis* that could be divided into 37 genetic types from A1 to A37 (Table 1). Group D contained 13 strains belonging to *B. pumilus*, with 7 genetic types, D1 to D7 (Table 1); Group F also contained 13 strains of 9 genetic types, F1 to F9 (Table 1), and belonging to *B. safensis*. In contrast, fewer bacteria were allotted into Group B, Group C and Group E and could not be assigned to any the described species due to low similarity. For example, the only strain in Group B showed 92.12%, 89.22% and 89.50% similarity with *B. altitudinis*, *B. pumilus* and *B. safensis* and a genetic distance of 0.079, 0.108 and 0.105, respectively. Both strains in Group C shared 91.04%, 90.21% and 91.02% similarity with the above type strains, with a genetic distance 0.09, 0.098 and 0.09, respectively. Similarly, the only member of Group E shared 89.48%, 91.41% and 93.93% similarity with the three type strains and a genetic distance of 0.105, 0.086 and 0.061, respectively. These unassigned strains represent novel bacterial taxa.

### Correlation between phylogenetic and geographic distribution

The geographical distribution of the 76 strains covered various marine environments: a subtropical coastal area, the Pacific Ocean, the Indian Ocean, the Arctic Ocean, the Atlantic and the South China Sea. 

Among these bacteria, those belonging to *B. altitudinis* were in the majority and had the widest geographical distribution; 48 of our isolates were allocated to this group in the concatenated gene tree (Group A in Figure 3). These isolates were mainly from three areas, the coastal area (○), South China Sea (□) and Pacific ocean (△), though some were isolated from the Indian Ocean (▽), the Atlantic Ocean (☆) and the Arctic Ocean. (◇). Group D contained twelve strains that were isolated from a Fujian coastal area and pelagic areas; however, no strain originated from the Arctic Ocean (◇) or South China Sea (□). The 12 strains in Group F were mainly from the coast area (○),South China Sea (□) and Arctic Ocean (◇). Among the above-mentioned special clades composed of putative novel species, two of three are from marine aquiculture environments, one from fish gut (strain 1 in Group B) and another from a shrimp farm (strain 70 in Group C).

In addition, according to the water depth, the habitats were arbitrarily divided into the upper layer (0-1000 m) and deep layer (>1000 m) and marked in green and black, respectively, in the phylogenetic tree (Figure 3). According to the tree, it was observed that the strains tended to cluster together to some extent according to the water depth. For example, in the largest group (Group A, *B. altitudinis*), bacteria from shallow areas tended to cluster together (in green). On the other hand, bacteria from the deep sea (in black) tended to cluster. Further, a principal component analysis (PCA) based on all strains was carried out to examine the key factors influencing their distribution using unweighted UniFrac. However, the correlation of the phylogenetic and geographic distribution was not significant (data not shown). This may be due to the inadequate strain numbers in other species.

**Figure 4:**
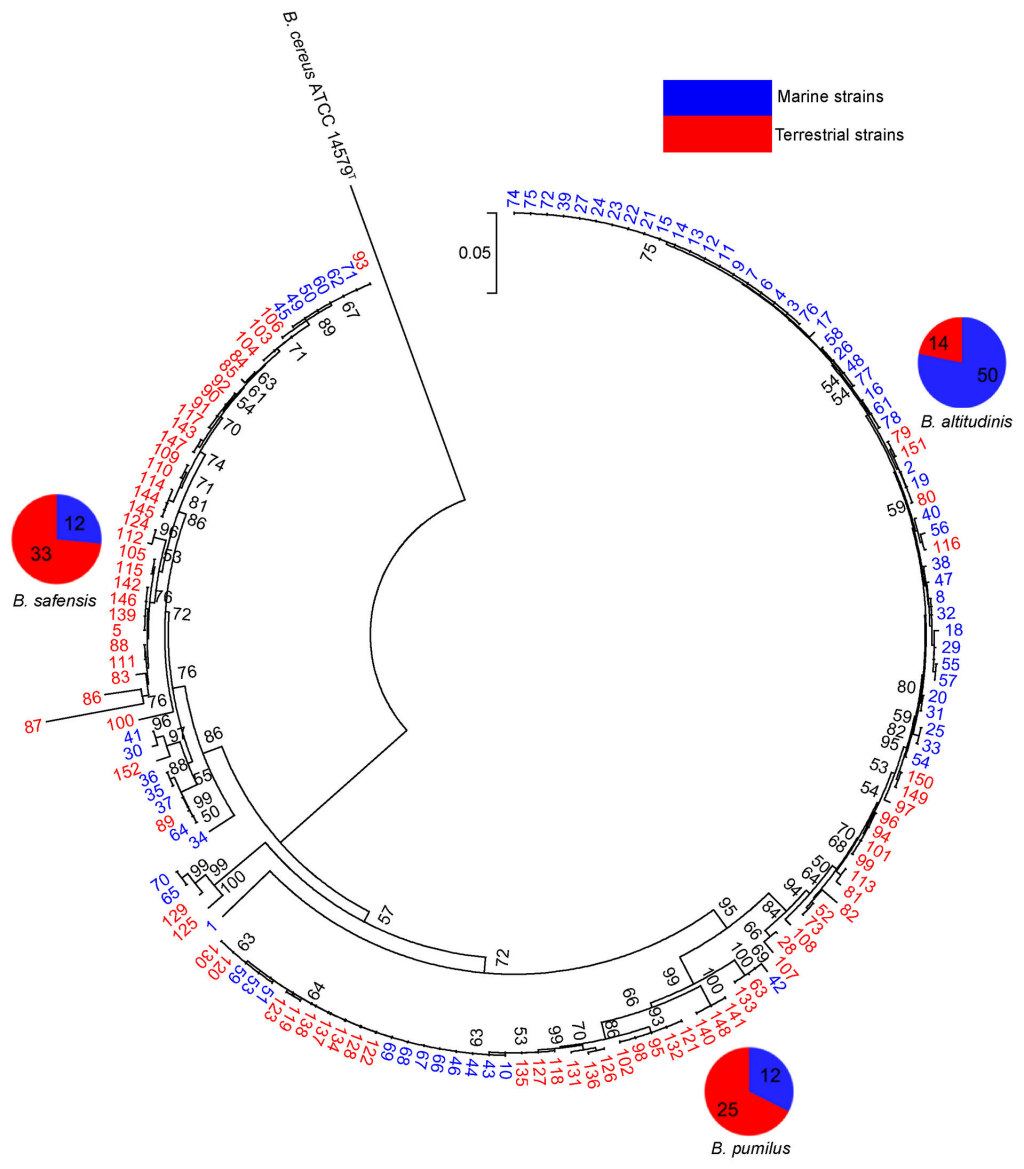
Phylogenetic tree based on *gyrB* genes of 152 strains of both marine and terrestrial origins. The tree was constructed using the neighbor-joining method with MEGA 5.0. Bootstrap values over 50% (1000 replications) were shown at each node. Bar, % estimated substitution. *B*. *cereus* ATCC 14579^T^ was used as the outgroup. The number represented the number of strains in each portion of the pie chart. The bacteria from marine environments were in blue, and the others were in red. The pie charts illustrated the proportions of marine and terrestrial origins in each large cluster.

To compare with their terrestrial counterparts, more sequences of the *gyrB* gene of the *B. pumilus* group were retrieved from GenBank (much less data for other housekeeping genes are available), and a phylogenetic tree of 152 strains was constructed (Figure 4). In general, the topological structure of the tree was the same as that constructed with our bacteria alone (Figure 3), though the three clades containing the potential novel species remain as minorities in the new tree. Some mistakes in nomenclature were observed for some strains retrieved from NCBI, such as strains 99, 101, 107, 108, 113 and 116, which actually belong to *B. altitudinis* rather than *B. pumilus*, as described in NCBI. 

Of note, based on the large tree of *gyrB* genes, the bacteria of marine origin tended to cluster together; with some exceptions, the strains of terrestrial origin also clustered together (Figure 4). Most of our marine isolates were placed in the large cluster of *B. altitudinis*, positioned as a separate clade (numbers in blue); a similar tendency was observed for the *B. pumilus* and *B. safensis* clusters. Most of the terrestrial bacteria were allotted to *B. safensis*, forming a distinct clade (in red). 

## Discussion

Many *Bacillus* strains have recently been isolated from marine environments, with bacteria of *B. pumilus* being frequently reported, in addition to *B. subtilis, B. licheniformis* and *B. cereus* [17,30-36]. Although the *B. altitudinis*, *B. pumilus* and *B. safensis* bacteria of the *B. pumilus* group cannot be differentiated by their 16S rRNA gene sequences, according to the data retrieved from PubMed, the bacteria from marine environments are generally placed in the *B. pumilus* group. To understand the diversity and systematic relationship of the bacteria in the *B. pumilus* group, we subjected 76 strains to MLSA based on seven housekeeping genes. Unexpectedly, most of our isolates actually belong to the species of *B. altitudinis* rather than *B. pumilus*. To our knowledge, this is the first report on the diversity and phylogeny of the *B. pumilus* group.

Our phylogenetic analysis showed that different housekeeping genes varied with regard to their discrimination resolution among the bacteria of the *B. pumilus* group. Among the seven housekeeping genes, the *pyrE* gene possessed, on average, the highest percentage of the polymorphic sites (29.30%) and the highest genetic distance (0.085), indicating that *pyrE* has the highest differentiation power. This was reconfirmed by the results of Ka/Ks ratios and intraspecies and interspecies similarity ranges. In addition, both *aroE* and *gyrB* also possesses a relative high resolution power. Considering the popularity of the *gyrB* gene in the GenBank database, we suggest *pyrE* and *gyrB* can be used as a standard marker to differentiate the closely related strains of the *B. pumilus* group. In the *B. subtilis* group, approximately 95% similarity of *gyrB* gene was accordant with 70% of DNA-DNA relatedness [15]. In other genera, for example, the *gyrB* gene also has been used as a marker to assign species. The genetic distance of the *gyrB* gene used to separate two species is 0.014, which was the equivalent of 70% DNA-DNA hybridization in *Micromonospora* species, as reported by Kasai et al. [36]. As another example, 0.02 genetic distance for the *gyrB* gene was used as a species boundary among the *Amycolatopsis* genus in a study by Everest et al. [37]. Similarly, Curtis et al. proposed using a genetic distance of 0.04 for five concatenated housekeeping genes to distinguish different species in *Kribbella* [38]. In the *B. pumilus* group, we found 95%-96% similarities of *gyrB* gene was the interspecies gap. Based on these results and further genome sequence data, we proposed three novel species of the genus *Bacillus*, represented by strain 1, 70 and 34. These bacteria shared low *gyrB* gene sequence similarity (89.50%-94.98%) with and large genetic distances (0.05-0.1) from the described type strains. The preliminary draft genome sequence analysis showed that their estimated DNA-DNA values (among 3 novel strains and 3 type strains) were below 70% (data unpublished), suggesting that they are potential novel species. Further phenotypic characterizations are needed to establish these bacteria as novel species.

The correlation analysis of phylogeny with geographical distribution indicated that the strains of Group A (*B. altitudinis*) were more widespread in marine environments than the other groups (Groups B-F) (Figure 3), suggests that Group A is adapted to a wide range of marine environments. Furthermore, our marine isolates tended to form clades corresponding to the water depth (Figure 3), and such distribution is in congruence with other reports. It has been shown that bacteria of *Exiguobacterium* tend to form genetic clusters by niche differentiation in water and sediment environments of the Cuatro Cienegas Basin [39]. As another example, Qian et al. found that there were significant differences in the diversity of microbial communities in the upper (2 and 50 m) and deeper layers (200 and 1500 m) of the Red sea, though there were no obvious differences within the same layer [40]. The distribution of bacteria is significantly influenced by environmental factors, such as salinity, temperature, oxygen and, in particular, water depth and pressure [41-43]. However, the mechanisms of ecological divergence require additional studies.

The phylogeny of the bacteria of diverse origins is shown in the expanded *gyrB* tree (Figure 4), reconfirming that the strains of *B. altitudinis* appear to be more widespread in marine environments, whereas the strains of *B. pumilus* and *B. safensis* tend to reside in terrestrial habitats. In fact, the type strain of *B. altitudinis*, which appeared randomly among our marine isolates, was isolated from an air sample from a high elevation (41 km), and a marine origin with seawater evaporation cannot be excluded. In the clade of *B. altitudinis*, the marine taxa appeared to have evolved from terrestrial taxa. So did a small marine branch (strains 45, 49, 50, 60, 62 and 71) in the *B. safensis* clade (Figure 4).

In summary, we analyzed the phylogeny of marine isolates closely related to the *B. pumilus* group using MLSA based on seven housekeeping genes. The bacteria of the *B. pumilus* group are frequently misnamed at the species level due to the high similarity in their 16S rRNA gene sequence. We found that both the *gyrB* and *pyrE* genes can be used as molecular marker to distinguish these closely related strains. Based on our MLSA results, we conclude that bacteria of *B. altitudinis* are most widely spread among the bacteria of the *B. pumilus* group in marine environment; while most bacteria from terrestrial habitats of this group actually belong to *B. safensis*. The results of this study provide the first report of the phylogenetic analysis of bacteria in this group and will help in the understanding of their ecological role, ecological evolution and adaptation to marine environments. However, the results based on MLSA are not enough to resolve these issues as the housekeeping genes used only occupy 0.1%~0.2% of the genome. Fingerprinting methods like RAPD, AFLP and Rep-PCR, and genome sequence analyses would differentiate them in more details. Currently, genome sequencing of 21 strains representing different branches of the *B. pumilus* group are undergoing, and further analyses will help to determine the taxonomic status of these species in this group, more important to gain insights into the evolution and adaption in marine environments.

## Supporting Information

File S1 **Supplementary material of Figure S1-S10 and Table S1-S8.**
(DOCX)
