# Supplementary material for: Phylogenetic Diversity of the Bacillus pumilus Group and the Marine Ecotype Revealed by Multilocus Sequence Analysis
Source: PLoS One. 2013 Nov 11;8(11):e80097. doi: 10.1371/journal.pone.0080097 (PMC3823796; doi:10.1371/journal.pone.0080097)
Supplement: File S1 — Supplementary material of Figure S1-S10 and Table S1-S8. (DOCX) [file pone.0080097.s001.docx]

**Supporting Information**

**Figure S1 The distribution map of the 76 strains from various marine environments.** Each red dot represents a strain, some dots overlapped.

**Figure S2 The interspecies gap in the *B. pumilus* group revealed by the number of strain pairs within different similarity grades of the housekeeping genes of 79 strains.**

**Figure S3 Ka/Ks of gene(s) in different species and all the tested strains of the *B. pumilus* group.**

**Figure S4 Phylogenetic tree based on *gyrB* gene.** The tree was constructed using the neighbor-joining method with MEGA 5.0. Bootstrap values over 50% (1000 replications) were shown at each node. Bar, % estimated substitution. *B. cereus* ATCC 14579^T^ was used as the outgroup.

**Figure S5 Phylogenetic tree based on *rpoB* gene.** The tree was constructed using the neighbor-joining method with MEGA 5.0. Bootstrap values over 50% (1000 replications) were shown at each node. Bar, % estimated substitution. *B. cereus* ATCC 14579^T^ was used as the outgroup.

**Figure S6 Phylogenetic tree based on *aroE* gene.** The tree was constructed using the neighbor-joining method with MEGA 5.0. Bootstrap values over 50% (1000 replications) were shown at each node. Bootstrap values over 50% (1000 replications) were shown at each node. Bar, % estimated substitution. *B. cereus* ATCC 14579^T^ was used as the outgroup.

**Figure S7 Phylogenetic tree based on *mutL* gene.** The tree was constructed using the neighbor-joining method with MEGA 5.0. Bootstrap values over 50% (1000 replications) were shown at each node. Bootstrap values over 50% (1000 replications) were shown at each node. Bar, % estimated substitution. *B. cereus* ATCC 14579^T^ was used as the outgroup.

**Figure S8** **Phylogenetic tree based on *pycA* gene.** The tree was constructed using the neighbor-joining method with MEGA 5.0. Bootstrap values over 50% (1000 replications) were shown at each node. Bar, % estimated substitution. *B. cereus* ATCC 14579^T^ was used as the outgroup.

**Figure S9 Phylogenetic tree based on *pyrE* gene.** The tree was constructed using the neighbor-joining method with MEGA 5.0. Bootstrap values over 50% (1000 replications) were shown at each node. Bar, % estimated substitution. *B. cereus* ATCC 14579^T^ was used as the outgroup.

**Figure S10 Phylogenetic tree based on *trpB* gene.** The tree was constructed using the neighbor-joining method with MEGA 5.0. Bootstrap values over 50% (1000 replications) were shown at each node. Bar, % estimated substitution. *B. cereus* ATCC 14579^T^ was used as the outgroup.

**Table S1 List of 73 strains retrieved from the NCBI database.**

**Table S2 Characteristics of the primers used in this study.**

**Table** **S3 GenBank accession numbers of all strains in this study.**

**Table S4 The similarity variation ranges of the house keeping genes of the 79 strains at intraspecies and interspecies levels.**

**Table S5 The correlation of genetic distance of the different housekeeping genes.**

**Table S6 The characteristics comparison of the single gene and concatenated genes in three species.**

**Table S7 The number of strain pairs within different similarity grades of the housekeeping genes of 79 strains.**

**Table S8 The Ka/Ks ratio of 7 single housekeeping genes in different species and all the tested strains of the *B. pumilus* group.**


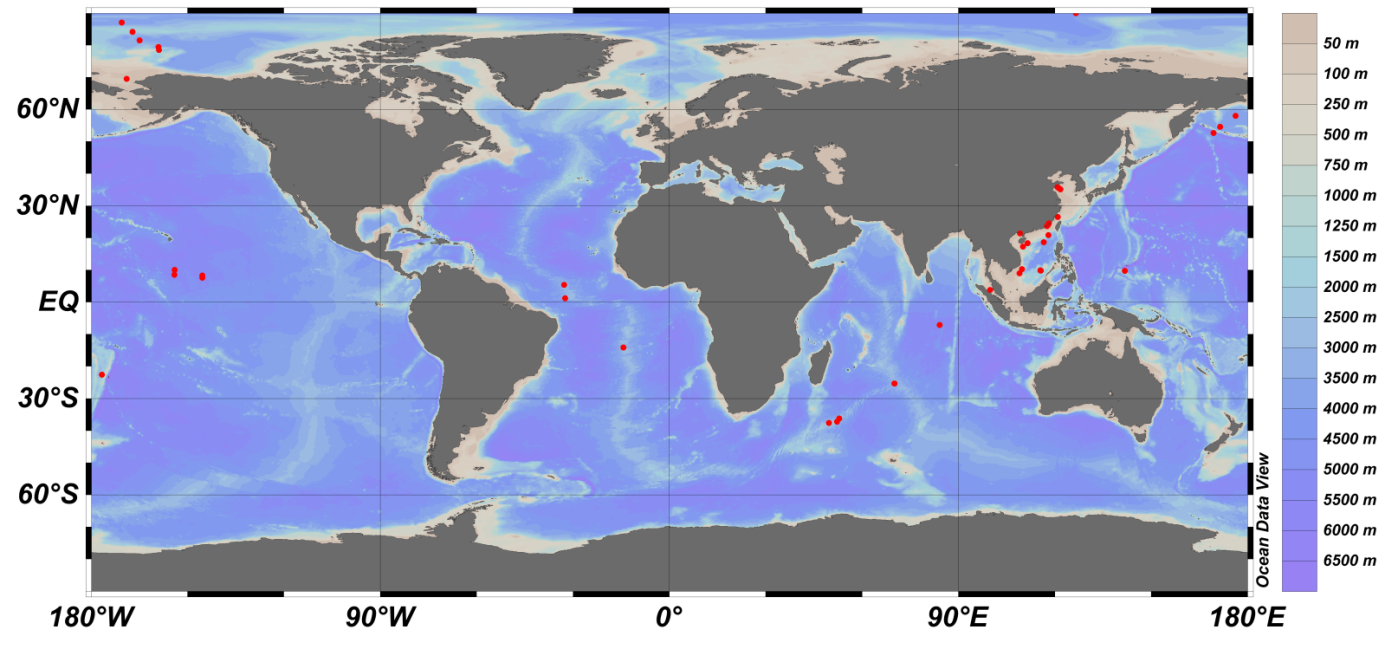


**Figure S1 The distribution map of the 76 strains from various marine environments.** Each red dot represents a strain, some dots overlapped.


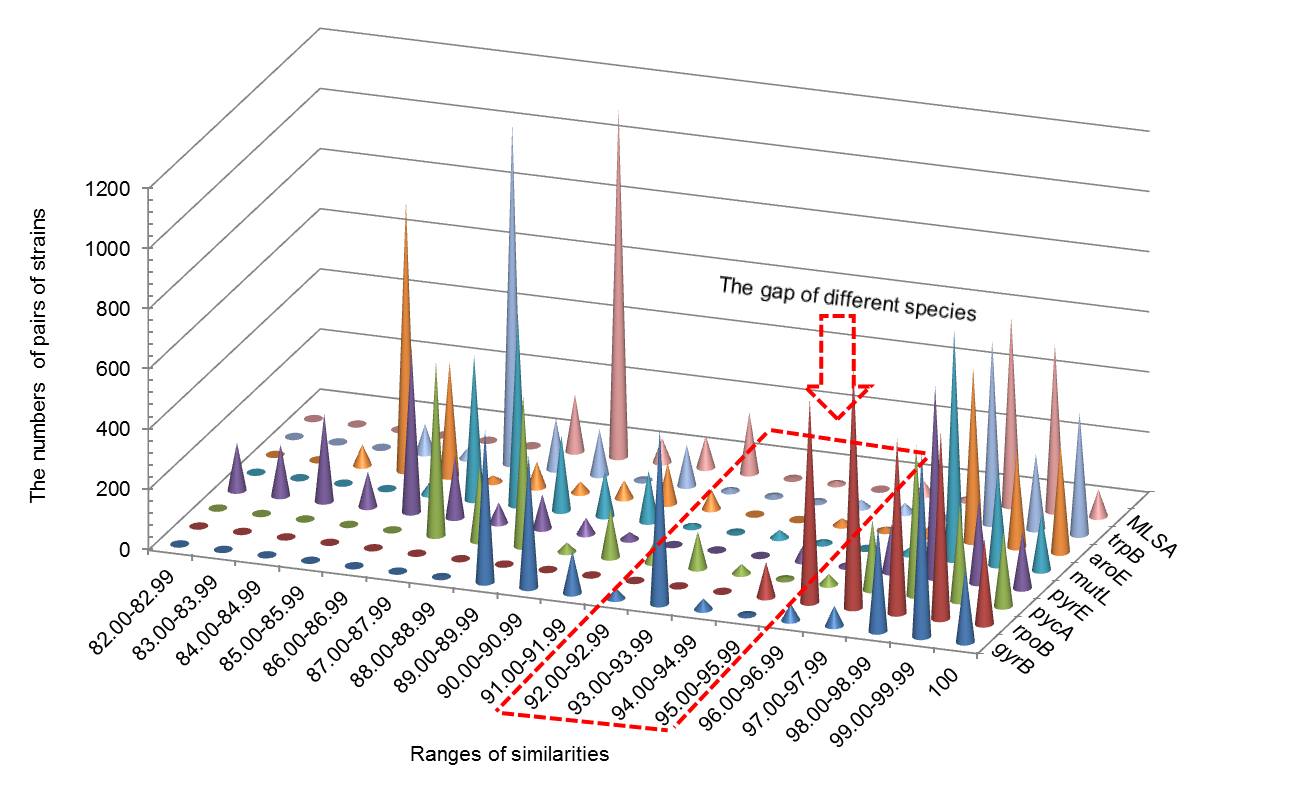


**Figure S2 The interspecies gap in the *B. pumilus* group revealed by the number of strain pairs within different similarity grades of the housekeeping genes of 79 strains**


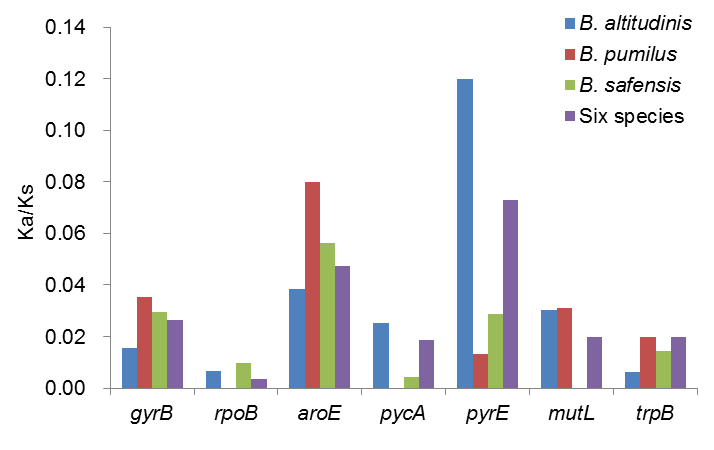


**Figure S3 Ka/Ks of gene(s) in different species and all the tested strains of the *B. pumilus* group**


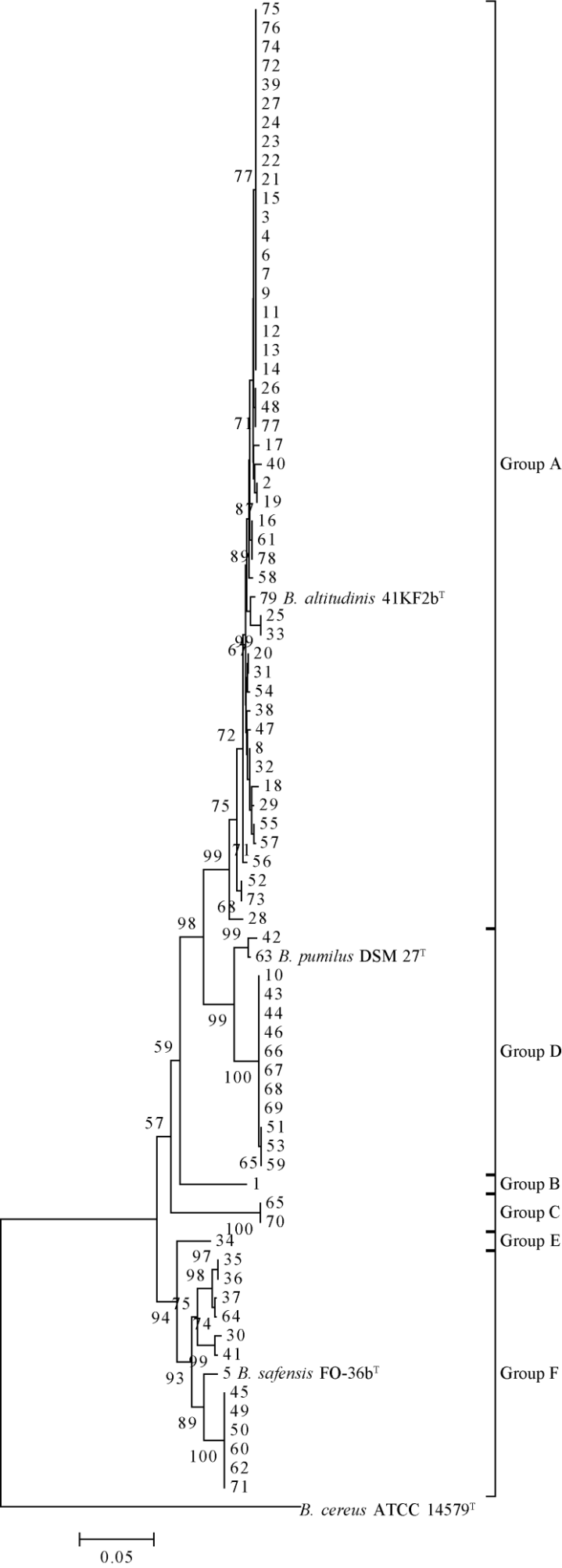


**Figure S4 Phylogenetic tree based on *gyrB* gene.** The tree was constructed using the neighbor-joining method with MEGA 5.0. Bootstrap values over 50% (1000 replications) were shown at each node. Bar, % estimated substitution. *B. cereus* ATCC 14579^T^ was used as the outgroup.


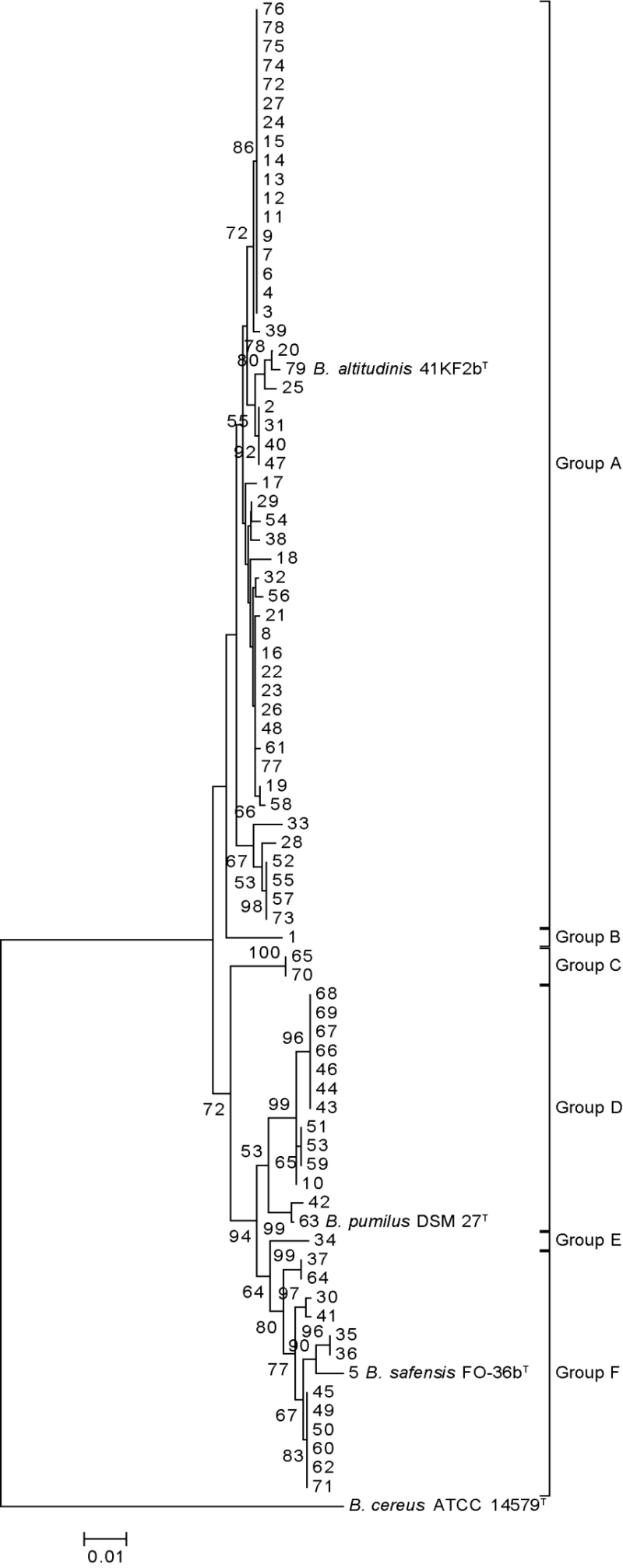


**Figure S5 Phylogenetic tree based on *rpoB* gene.** The tree was constructed using the neighbor-joining method with MEGA 5.0. Bootstrap values over 50% (1000 replications) were shown at each node. Bar, % estimated substitution. *B. cereus* ATCC 14579^T^ was used as the outgroup.


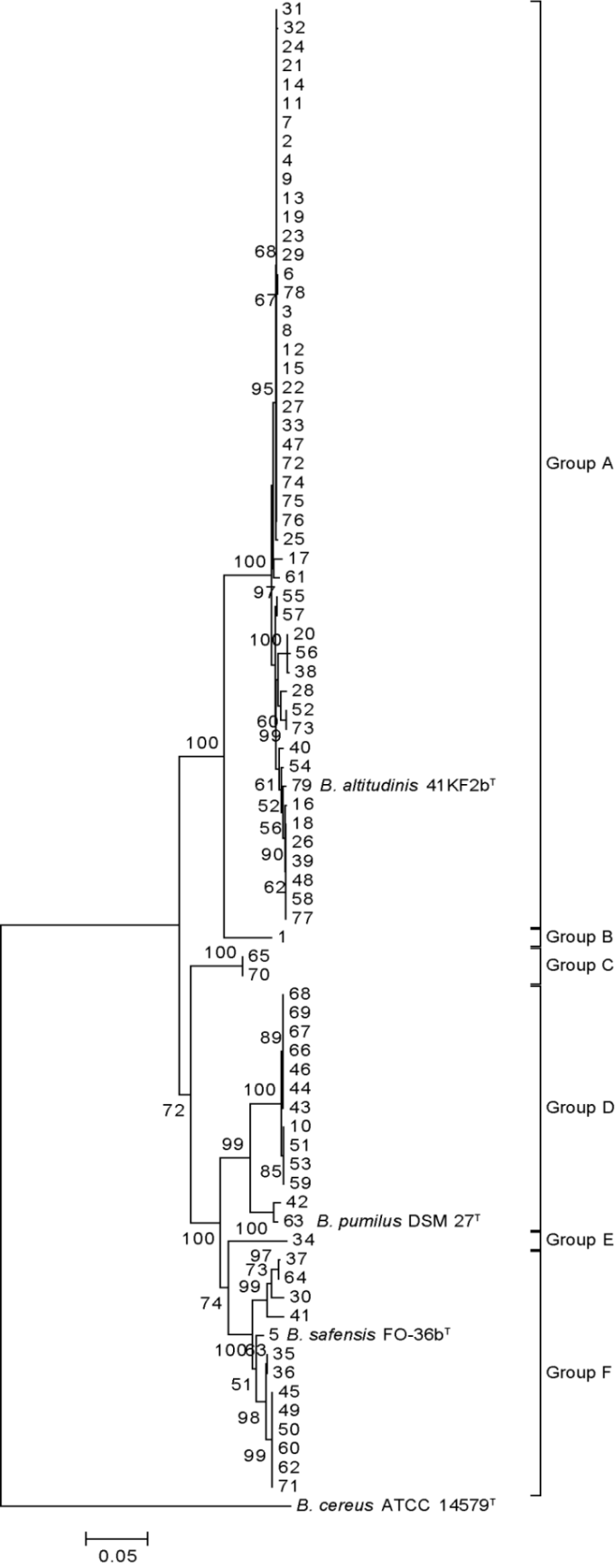


**Figure S6 Phylogenetic tree based on *aroE* gene.** The tree was constructed using the neighbor-joining method with MEGA 5.0. Bootstrap values over 50% (1000 replications) were shown at each node. Bootstrap values over 50% (1000 replications) were shown at each node. Bar, % estimated substitution. *B. cereus* ATCC 14579^T^ was used as the outgroup.


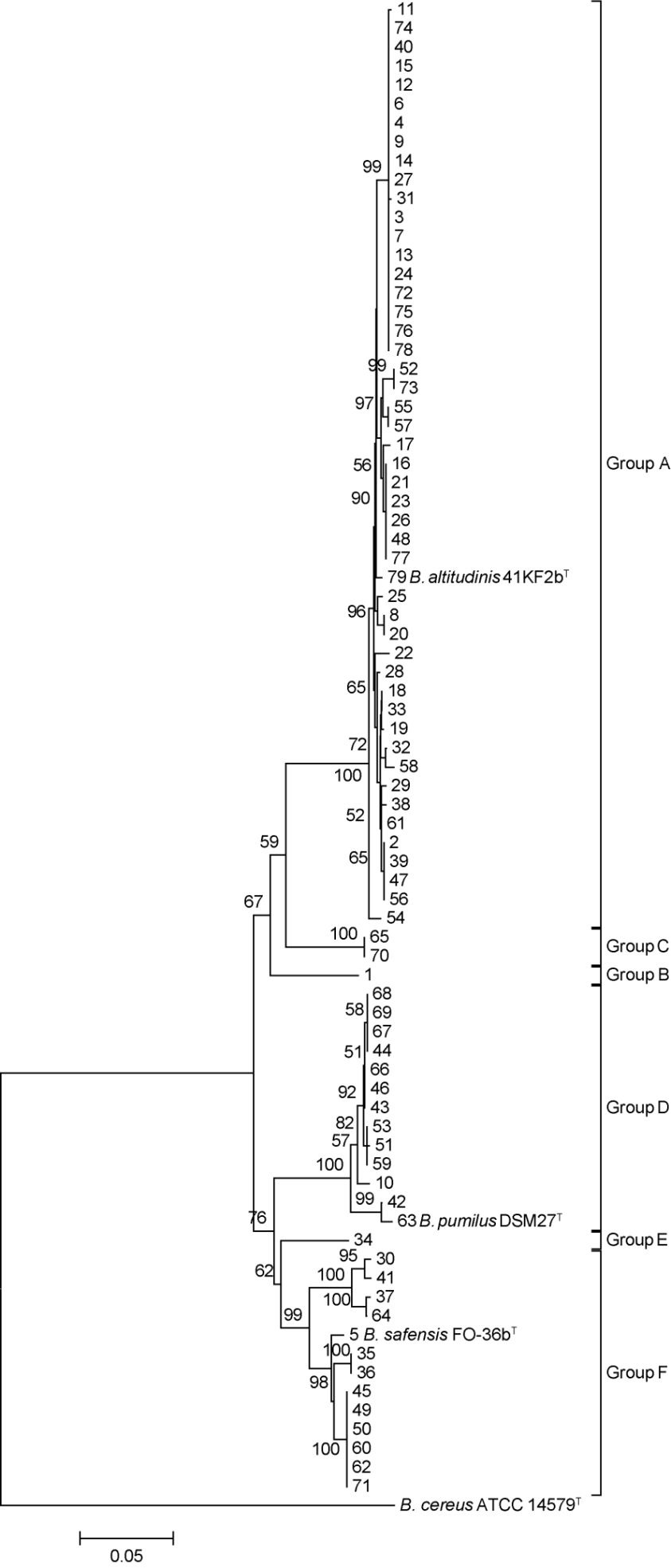


**Figure S7 Phylogenetic tree based on *mutL* gene.** The tree was constructed using the neighbor-joining method with MEGA 5.0. Bootstrap values over 50% (1000 replications) were shown at each node. Bootstrap values over 50% (1000 replications) were shown at each node. Bar, % estimated substitution. *B. cereus* ATCC 14579^T^ was used as the outgroup.


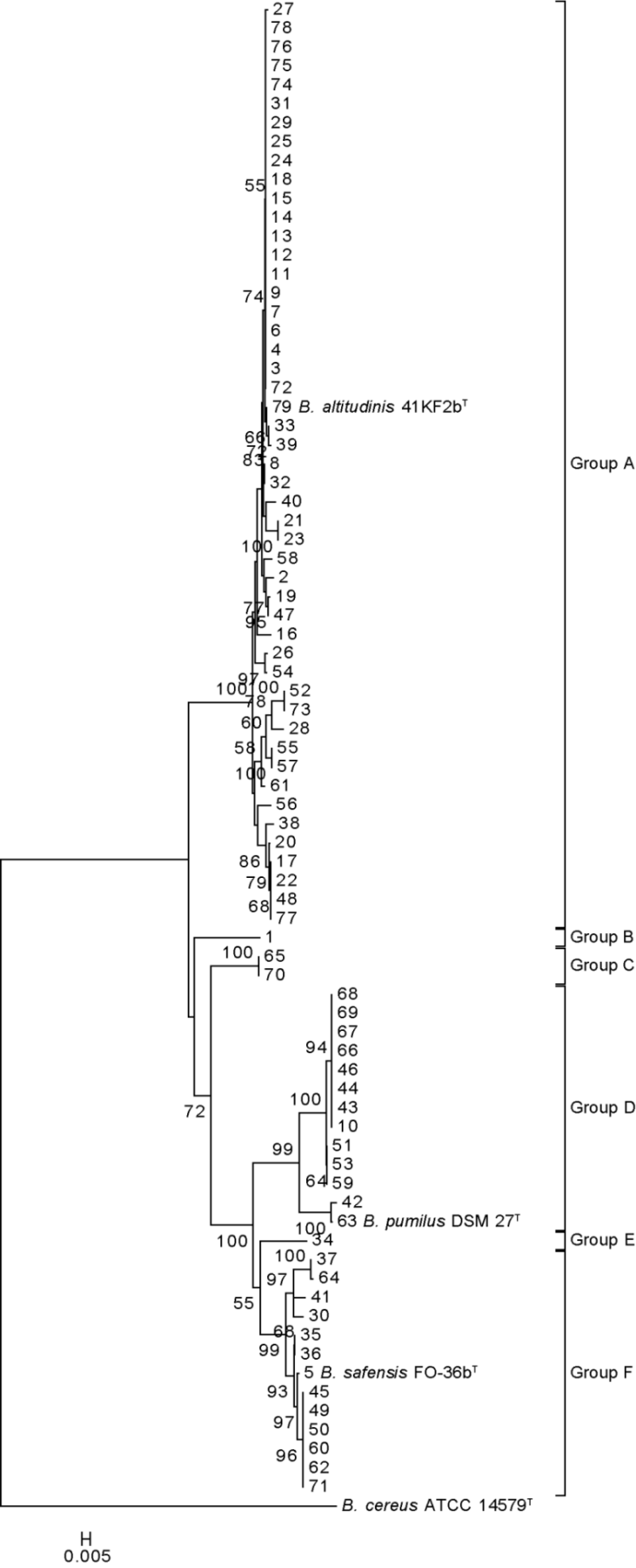


**Figure S8** **Phylogenetic tree based on *pycA* gene.** The tree was constructed using the neighbor-joining method with MEGA 5.0. Bootstrap values over 50% (1000 replications) were shown at each node. Bar, % estimated substitution. *B. cereus* ATCC 14579^T^ was used as the outgroup.


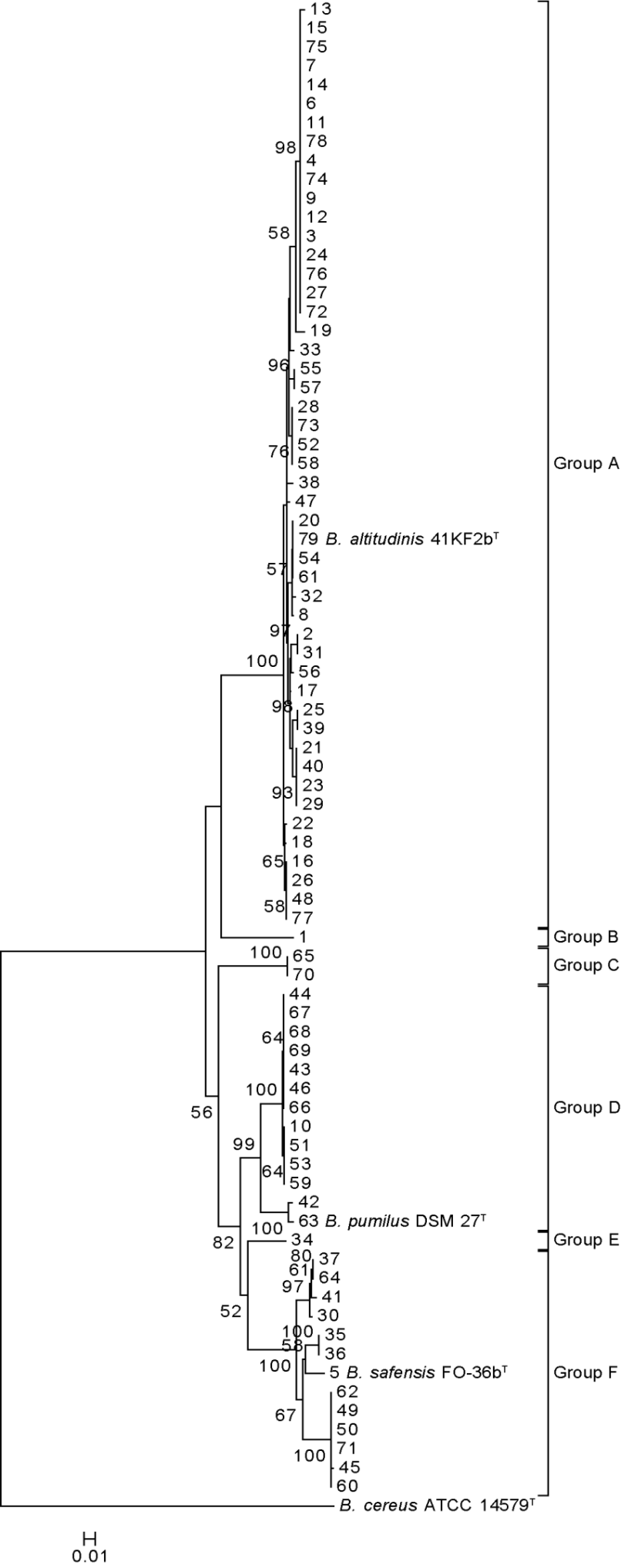


**Figure S9 Phylogenetic tree based on *pyrE* gene.** The tree was constructed using the neighbor-joining method with MEGA 5.0. Bootstrap values over 50% (1000 replications) were shown at each node. Bar, % estimated substitution. *B. cereus* ATCC 14579^T^ was used as the outgroup.


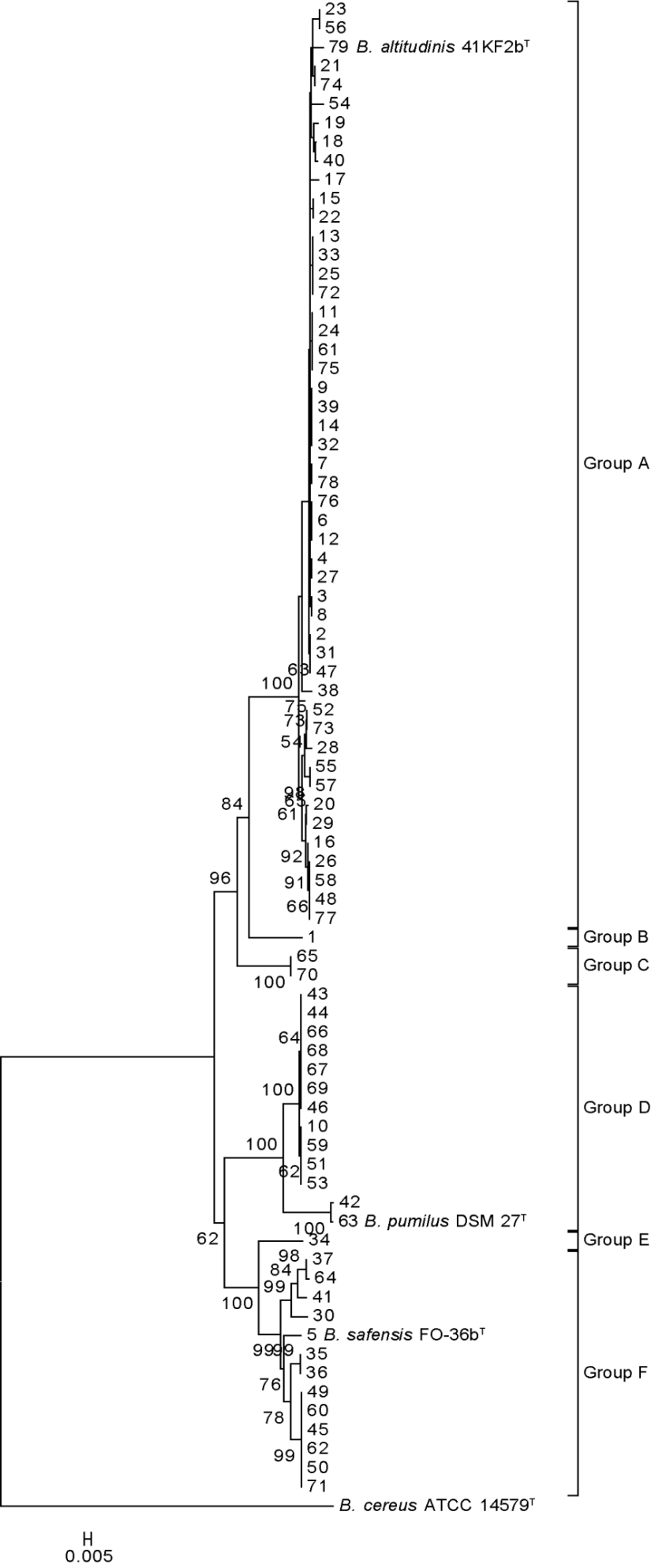


**Figure S10 Phylogenetic tree based on *trpB* gene.** The tree was constructed using the neighbor-joining method with MEGA 5.0. Bootstrap values over 50% (1000 replications) were shown at each node. Bar, % estimated substitution. *B. cereus* ATCC 14579^T^ was used as the outgroup.

**Table S1 List of 73 strains retrieved from the NCBI database**

| Sequential No. | Original No. | Species | Origin | Region | 16S rDNA accession | *gyrB* accession |
| --- | --- | --- | --- | --- | --- | --- |
| 80 | Se1 | *B. altitudinis* | Seeds | Vitis vinifera L. cv. Zweigelt | HQ432811 | HQ597038 |
| 81 | F3 | *B. pumilus* | Flowers | Vitis vinifera L. cv. Zweigelt | EU285662 | HQ597035 |
| 82 | AF35 | *B. aerophilus* | Oil reservoirs | The Campos Basin Brazil | JQ183032 | JQ183054 |
| 83 | SG10 | *B. safensis* | Oil reservoirs | The Campos Basin Brazil | - | JQ183053 |
| 84 | SG1 | *B. safensis* | Oil reservoirs | The Campos Basin Brazil | - | JQ183055 |
| 85 | SG5 | *B. safensis* | Oil reservoirs | The Campos Basin Brazil | JQ183021 | JQ183057 |
| 86 | SG54-1 | *B. safensis* | Oil reservoirs | The Campos Basin Brazil | - | JQ183059 |
| 87 | CBMAI:971:SG29 | *B. safensis* | Formation water | E. Santos-Neto Brazil | - | JX499918 |
| 88 | CBMAI:967:SG13 | *B. safensis* | Formation water | E. Santos-Neto Brazil | - | JX499915 |
| 89 | Bp1 | *B. pumilus* | - | - | JX188064 | JX183188 |
| 90 | Bp2 | *B. pumilus* | - | - | JX183146 | JX183189 |
| 91 | Bp3 | *B. pumilus* | - | - | JN699031 | JX183190 |
| 92 | Bp5 | *B. pumilus* | - | - | JN699030 | JX183191 |
| 93 | Bp6 | *B. pumilus* | - | - | JX183147 | JX183192 |
| 94 | Bp7 | *B. pumilus* | - | - | JN699029 | JX183193 |
| 95 | Bp8 | *B. pumilus* | - | - | JN699028 | JX183194 |
| 96 | Bp9 | *B. pumilus* | - | - | JX183148 | JX183195 |
| 97 | Bp11 | *B. pumilus* | - | - | JN699027 | JX183196 |
| 98 | Bp12 | *B. pumilus* | - | - | JN699026 | JX183197 |
| 99 | Bp13 | *B. pumilus* | - | - | JX183149 | JX183198 |
| 100 | Bp14 | *B. pumilus* | - | - | JX183150 | JX183199 |
| 101 | Bp15 | *B. pumilus* | - | - | JN699025 | JX183200 |
| 102 | Bp16 | *B. pumilus* | - | - | JN699024 | JX183201 |
| 103 | Bp17 | *B. pumilus* | - | - | JX183151 | JX183202 |
| 104 | Bp18 | *B. pumilus* | - | - | JN699023 | JX183203 |
| 105 | Bp19 | *B. pumilus* | - | - | JN699022 | JX183204 |
| 106 | Bp20 | *B. pumilus* | - | - | JX183152 | JX183205 |
| 107 | Bp21 | *B. pumilus* | - | - | JX987053 | JX183206 |
| 108 | Bp22 | *B. pumilus* | - | - | JX183154 | JX183207 |
| 109 | Bp23 | *B. pumilus* | - | - | JX183155 | JX183208 |
| 110 | Bp24 | *B. pumilus* | - | - | JX183156 | JX183209 |
| 111 | Bp25 | *B. pumilus* | - | - | JX183157 | JX183210 |
| 112 | Bp26 | *B. pumilus* | - | - | JX183158 | JX183211 |
| 113 | Bp27 | *B. pumilus* | - | - | JN699021 | JX183212 |
| 114 | Bp29 | *B. pumilus* | - | - | JN699020 | JX183213 |
| 115 | Bp30 | *B. pumilus* | - | - | - | JX183214 |
| 116 | Bp31 | *B. pumilus* | - | - | - | JX183215 |
| 117 | AUEC29 | *B. pumilus* | Plant rhizosphere soil | Tibet China | HM585071 | HM585095 |
| 118 | AUEM104 | *B. pumilus* | Plant rhizosphere soil | Tibet China | HM585069 | HM585093 |
| 119 | AUES82 | *B. pumilus* | Plant rhizosphere soil | Tibet China | HM585067 | HM585091 |
| 120 | BKS1-108 | *B. pumilus* | Plant rhizosphere soil | Tibet China | HM585061 | HM585085 |
| 121 | KYC18 | *B. pumilus* | Plant rhizosphere soil | Tibet China | HM585057 | HM585081 |
| 122 | AUEM12 | *B. pumilus* | Plant rhizosphere soil | Tibet China | HM585070 | HM585094 |
| 123 | AUES51 | *B. safensis* | Plant rhizosphere soil | Tibet China | HM585068 | HM585092 |
| 124 | HTC38 | *B. pumilus* | Plant rhizosphere soil | Tibet China | HM585064 | HM585088 |
| 125 | BKS2-67 | *B. safensis* | Plant rhizosphere soil | Tibet China | HM585060 | HM585084 |
| 126 | KYC24 | *B. pumilus* | plant rhizosphere soil | Tibet China | HM585056 | HM585080 |
| 127 | GBSC66 | *B. pumilus* | Plant rhizosphere soil | Tibet China | GU568205 | GU568235 |
| 128 | LLTC93 | *B. safensis* | plant rhizosphere soil | Tibet China | GU568199 | GU568229 |
| 129 | LNXM12 | *B.pumilus* | Plant rhizosphere soil | Tibet China | GU568198 | GU568227 |
| 130 | LNXM65 | *B. safensis* | Plant rhizosphere soil | Tibet China | GU568195 | GU568225 |
| 131 | NMCC46 | *B. pumilus* | Plant rhizosphere soil | Tibet China | GU568191 | GU568221 |
| 132 | NMSW10 | *B. pumilus* | Plant rhizosphere soil | Tibet China | GU568189 | GU568219 |
| 133 | GBSW2 | *B. pumilus* | Plant rhizosphere soil | Tibet China | GU568204 | GU568234 |
| 134 | GBSW19 | *B. pumilus* | Plant rhizosphere soil | Tibet China | GU568202 | GU568232 |
| 135 | YBWC18 | *B. pumilus* | Plant rhizosphere soil | Tibet China | GU568179 | GU568228 |
| 136 | MZGC1 | *B. pumilus* | Plant rhizosphere soil | Tibet China | GU568192 | GU568222 |
| 137 | NMTD17 | *B. pumilus* | Plant rhizosphere soil | Tibet China | GU568184 | GU568214 |
| 138 | FO-038 | *B. pumilus* | Clean-room airlock | California US | AF234856 | AY167866 |
| 139 | SAFN-034 | *B. pumilus* | Clean-room airlock | California US | AY167882 | AY167874 |
| 140 | SAFN-029 | *B. safensis* | Clean-room airlock | California US | AY167883 | AY167875 |
| 141 | SAFN-001 | *B. safensis* | Entrance ﬂoor | California US | AY167886 | AY167877 |
| 142 | SAFN-027 | *B. safensis* | Anteroom | California US | AY167884 | AY167876 |
| 143 | SAFN-036 | *B. safensis* | Clean-room ﬂoor | California US | AY167881 | AY167873 |
| 144 | SAFN-037 | *B. safensis* | Clean-room ﬂoor | California US | AY167880 | AY167872 |
| 145 | FO-033 | *B. safensis* | Clean-room air particulate | California US | AF234851 | AY167868 |
| 146 | KL-052 | *B. safensis* | Clean-room cabinet top | California US | AY030327 | AY167878 |
| 147 | ATCC 27142 | *B. pumilus* | spacecraft and assembly-facility surfaces | California US | AY876287 | AY167870 |
| 148 | SAFR-032 | *B. pumilus* | Clean-room airlock | California US | AY167879 | AY167871 |
| 149 | S-1 | *B. pumilus* | - | - | AGBY01000000 | AGBY01000000 |
| 150 | KACC 16563 | *B. aerophilus* | Soil | Greenhouse at the University of Seoul | NC_017743 | NC_017743 |
| 151 | BA06 | *B. pumilus* | The proteinaceous soil | - | AMDH01000000 | AMDH01000000 |
| 152 | Biosubtyl | *B. pumilus* | - | - | JN580980 | JN575340 |

“-”: The information is unknown

**Table S2** **Characteristics of the primers used in this study**

| Gene | Produce name | Primer name | Sequence (5' to 3') | Size (bp) | Annealing temperature (°C) |
| --- | --- | --- | --- | --- | --- |
| 16S rDNA | rRNA | 27F | AGAGTTTGATCCTGGCTCAG | 1473 | 55 |
|  |  | 1492R | ACGGCTACCTTGTTACGACT |  |  |
| *gyrB* | Gyrase B subunit | gyrBF | TTATCTACGACCTTAGACG | 1045 | 55 |
|  |  | gyrBR | TAAATTGAAGTCTTCTCCG |  |  |
| *rpoB* | RNA polymerase *β* subunit | rpoBF | GTTGGCTTCATGACTTGGGA | 1041 | 55 |
|  |  | rpoBR | ACGTTCCATACCTAAACTTTG |  |  |
| *aroE* | Shikimate 5-dehydrogenase | aroEF | CATAGATCAGTGATGTTT | 818 | 51 |
|  |  | aroER | TCAATGTGTTCAAAGAAATT |  |  |
| *mutL* | DNA mismatch repair protein | mutLF | TGAAGTTCCTGCTCTTTACT | 894 | 45 |
|  |  | mutLR | TATTCAGTTATCCGATGACCT |  |  |
| *trpB* | Tryptophan synthase subunit beta | trpBF | ATGTACGCATATCCAAATGA | 949 | 55 |
|  |  | trpBR | GTGGCACTCACATATTGAAC |  |  |
| *pyrE* | Orotate phosphoribosyltransferase | pyrBF | AGACCGTTTCTTCCATCCA | 577 | 57 |
|  |  | pyrBR | CACCTATTACAAATCAAAGC |  |  |
| *pycA* | Pyruvate carboxylase A | pycAF | GATTTAATGCTTTCATCCTTA | 909 | 55 |
|  |  | pycAR | AATGGACTATTCACCTATGC |  |  |

**Table S3 GenBank accession numbers of all strains in this study**

| Sequential No | 16S rDNA | *gyrB* | *rpoB* | *aroE* | *mutL* | *pycA* | *pyrE* | *trpB* |
| --- | --- | --- | --- | --- | --- | --- | --- | --- |
| 1 | JX680066 | JX680142 | JX679987 | KC346447 | KC346526 | KC346605 | KC346684 | KC346763 |
| 2 | JX680067 | JX680143 | JX679988 | KC346448 | KC346527 | KC346606 | KC346685 | KC346764 |
| 3 | JX680068 | JX680144 | JX679989 | KC346449 | KC346528 | KC346607 | KC346686 | KC346765 |
| 4 | JX680069 | JX680145 | JX679990 | KC346450 | KC346529 | KC346608 | KC346687 | KC346766 |
| 5 | KC346444 | JX680146 | JX679991 | KC346451 | KC346530 | KC346609 | KC346688 | KC346767 |
| 6 | JX680070 | JX680147 | JX679992 | KC346452 | KC346531 | KC346610 | KC346689 | KC346768 |
| 7 | JX680071 | JX680148 | JX679993 | KC346453 | KC346532 | KC346611 | KC346690 | KC346769 |
| 8 | JX680072 | JX680149 | JX679994 | KC346454 | KC346533 | KC346612 | KC346691 | KC346770 |
| 9 | JX680073 | JX680150 | JX679995 | KC346455 | KC346534 | KC346613 | KC346692 | KC346771 |
| 10 | JX680074 | JX680151 | JX679996 | KC346456 | KC346535 | KC346614 | KC346693 | KC346772 |
| 11 | JX680075 | JX680152 | JX679997 | KC346457 | KC346536 | KC346615 | KC346694 | KC346773 |
| 12 | JX680076 | JX680153 | JX679998 | KC346458 | KC346537 | KC346616 | KC346695 | KC346774 |
| 13 | JX680077 | JX680154 | JX679999 | KC346459 | KC346538 | KC346617 | KC346696 | KC346775 |
| 14 | JX680078 | JX680155 | JX680000 | KC346460 | KC346539 | KC346618 | KC346697 | KC346776 |
| 15 | JX680079 | JX680156 | JX680001 | KC346461 | KC346540 | KC346619 | KC346698 | KC346777 |
| 16 | JX680080 | JX680157 | JX680002 | KC346462 | KC346541 | KC346620 | KC346699 | KC346778 |
| 17 | JX680081 | JX680158 | JX680003 | KC346463 | KC346542 | KC346621 | KC346700 | KC346779 |
| 18 | JX680082 | JX680159 | JX680004 | KC346464 | KC346543 | KC346622 | KC346701 | KC346780 |
| 19 | JX680083 | JX680160 | JX680005 | KC346465 | KC346544 | KC346623 | KC346702 | KC346781 |
| 20 | JX680084 | JX680161 | JX680006 | KC346466 | KC346545 | KC346624 | KC346703 | KC346782 |
| 21 | JX680085 | JX680162 | JX680007 | KC346467 | KC346546 | KC346625 | KC346704 | KC346783 |
| 22 | JX680086 | JX680163 | JX680008 | KC346468 | KC346547 | KC346626 | KC346705 | KC346784 |
| 23 | JX680087 | JX680164 | JX680009 | KC346469 | KC346548 | KC346627 | KC346706 | KC346785 |
| 24 | JX680088 | JX680165 | JX680010 | KC346470 | KC346549 | KC346628 | KC346707 | KC346786 |
| 25 | JX680089 | JX680166 | JX680011 | KC346471 | KC346550 | KC346629 | KC346708 | KC346787 |
| 26 | JX680090 | JX680167 | JX680012 | KC346472 | KC346551 | KC346630 | KC346709 | KC346788 |
| 27 | JX680091 | JX680168 | JX680013 | KC346473 | KC346552 | KC346631 | KC346710 | KC346789 |
| 28 | JX680092 | JX680169 | JX680014 | KC346474 | KC346553 | KC346632 | KC346711 | KC346790 |
| 29 | JX680093 | JX680170 | JX680015 | KC346475 | KC346554 | KC346633 | KC346712 | KC346791 |
| 30 | JX680094 | JX680171 | JX680016 | KC346476 | KC346555 | KC346634 | KC346713 | KC346792 |
| 31 | JX680095 | JX680172 | JX680017 | KC346477 | KC346556 | KC346635 | KC346714 | KC346793 |
| 32 | JX680096 | JX680173 | JX680018 | KC346478 | KC346557 | KC346636 | KC346715 | KC346794 |
| 33 | JX680097 | JX680174 | JX680019 | KC346479 | KC346558 | KC346637 | KC346716 | KC346795 |
| 34 | JX680098 | JX680175 | JX680020 | KC346480 | KC346559 | KC346638 | KC346717 | KC346796 |
| 35 | JX680099 | JX680176 | JX680021 | KC346481 | KC346560 | KC346639 | KC346718 | KC346797 |
| 36 | JX680100 | JX680177 | JX680022 | KC346482 | KC346561 | KC346640 | KC346719 | KC346798 |
| 37 | JX680101 | JX680178 | JX680023 | KC346483 | KC346562 | KC346641 | KC346720 | KC346799 |
| 38 | JX680102 | JX680179 | JX680024 | KC346484 | KC346563 | KC346642 | KC346721 | KC346800 |
| 39 | JX680103 | JX680180 | JX680025 | KC346485 | KC346564 | KC346643 | KC346722 | KC346801 |
| 40 | JX680104 | JX680181 | JX680026 | KC346486 | KC346565 | KC346644 | KC346723 | KC346802 |
| 41 | JX680105 | JX680182 | JX680027 | KC346487 | KC346566 | KC346645 | KC346724 | KC346803 |
| 42 | JX680106 | JX680183 | JX680028 | KC346488 | KC346567 | KC346646 | KC346725 | KC346804 |
| 43 | JX680107 | JX680184 | JX680029 | KC346489 | KC346568 | KC346647 | KC346726 | KC346805 |
| 44 | JX680108 | JX680185 | JX680030 | KC346490 | KC346569 | KC346648 | KC346727 | KC346806 |
| 45 | JX680109 | JX680186 | JX680031 | KC346491 | KC346570 | KC346649 | KC346728 | KC346807 |
| 46 | JX680110 | JX680187 | JX680032 | KC346492 | KC346571 | KC346650 | KC346729 | KC346808 |
| 47 | JX680111 | JX680188 | JX680033 | KC346493 | KC346572 | KC346651 | KC346730 | KC346809 |
| 48 | JX680112 | JX680189 | JX680034 | KC346494 | KC346573 | KC346652 | KC346731 | KC346810 |
| 49 | JX680113 | JX680190 | JX680035 | KC346495 | KC346574 | KC346653 | KC346732 | KC346811 |
| 50 | JX680114 | JX680191 | JX680036 | KC346496 | KC346575 | KC346654 | KC346733 | KC346812 |
| 51 | JX680115 | JX680192 | JX680037 | KC346497 | KC346576 | KC346655 | KC346734 | KC346813 |
| 52 | JX680116 | JX680193 | JX680038 | KC346498 | KC346577 | KC346656 | KC346735 | KC346814 |
| 53 | JX680117 | JX680194 | JX680039 | KC346499 | KC346578 | KC346657 | KC346736 | KC346815 |
| 54 | JX680118 | JX680195 | JX680040 | KC346500 | KC346579 | KC346658 | KC346737 | KC346816 |
| 55 | JX680119 | JX680196 | JX680041 | KC346501 | KC346580 | KC346659 | KC346738 | KC346817 |
| 56 | JX680120 | JX680197 | JX680042 | KC346502 | KC346581 | KC346660 | KC346739 | KC346818 |
| 57 | JX680121 | JX680198 | JX680043 | KC346503 | KC346582 | KC346661 | KC346740 | KC346819 |
| 58 | JX680122 | JX680199 | JX680044 | KC346504 | KC346583 | KC346662 | KC346741 | KC346820 |
| 59 | JX680123 | JX680200 | JX680045 | KC346505 | KC346584 | KC346663 | KC346742 | KC346821 |
| 60 | JX680124 | JX680201 | JX680046 | KC346506 | KC346585 | KC346664 | KC346743 | KC346822 |
| 61 | JX680125 | JX680202 | JX680047 | KC346507 | KC346586 | KC346665 | KC346744 | KC346823 |
| 62 | JX680126 | JX680203 | JX680048 | KC346508 | KC346587 | KC346666 | KC346745 | KC346824 |
| 63 | KC346445 | JX680220 | JX680065 | KC346509 | KC346588 | KC346667 | KC346746 | KC346825 |
| 64 | JX680127 | JX680204 | JX680049 | KC346510 | KC346589 | KC346668 | KC346747 | KC346826 |
| 65 | JX680128 | JX680205 | JX680050 | KC346511 | KC346590 | KC346669 | KC346748 | KC346827 |
| 66 | JX680129 | JX680206 | JX680051 | KC346512 | KC346591 | KC346670 | KC346749 | KC346828 |
| 67 | JX680130 | JX680207 | JX680052 | KC346513 | KC346592 | KC346671 | KC346750 | KC346829 |
| 68 | JX680131 | JX680208 | JX680053 | KC346514 | KC346593 | KC346672 | KC346751 | KC346830 |
| 69 | JX680132 | JX680209 | JX680054 | KC346515 | KC346594 | KC346673 | KC346752 | KC346831 |
| 70 | JX680133 | JX680210 | JX680055 | KC346516 | KC346595 | KC346674 | KC346753 | KC346832 |
| 71 | JX680134 | JX680211 | JX680056 | KC346517 | KC346596 | KC346675 | KC346754 | KC346833 |
| 72 | JX680135 | JX680212 | JX680057 | KC346518 | KC346597 | KC346676 | KC346755 | KC346834 |
| 73 | JX680136 | JX680213 | JX680058 | KC346519 | KC346598 | KC346677 | KC346756 | KC346835 |
| 74 | JX680137 | JX680214 | JX680059 | KC346520 | KC346599 | KC346678 | KC346757 | KC346836 |
| 75 | JX680138 | JX680215 | JX680060 | KC346521 | KC346600 | KC346679 | KC346758 | KC346837 |
| 76 | JX680139 | JX680216 | JX680061 | KC346522 | KC346601 | KC346680 | KC346759 | KC346838 |
| 77 | JX680140 | JX680217 | JX680062 | KC346523 | KC346602 | KC346681 | KC346760 | KC346839 |
| 78 | JX680141 | JX680218 | JX680063 | KC346524 | KC346603 | KC346682 | KC346761 | KC346840 |
| 79 | KC346446 | JX680219 | JX680064 | KC346525 | KC346604 | KC346683 | KC346762 | KC346841 |

**Table S4 The similarity variation ranges of the house keeping genes of the 79 strains at intraspecies and interspecies levels**

|  | Similarities (%) | |
| --- | --- | --- |
|  | Intraspecies | Interspecies |
| 16S rDNA | 99.6-100 | 99.5-100 |
| *gyrB* | 96.1-100 | 89.0-96.0 |
| *rpoB* | 97.7-100 | 95.7-98.5 |
| *pycA* | 95.9-100 | 87.2-95.1 |
| *pyrE* | 95.2-100 | 82.1-91.6 |
| *mutL* | 94.6-100 | 86.5-93.1 |
| *aroE* | 95.0-100 | 84.1-92.6 |
| *trpB* | 95.0-100 | 85.1-94.0 |
| Seven concatenated genes | 96.3-100 | 88.5-94.2 |

**Table S5 The correlation of genetic distance of the different housekeeping genes**

|  | 16S rDNA | *gyrB* | *rpoB* | *aroE* | *mutL* | *pycA* | *pyrE* | *prpB* | 7 concatenated genes |
| --- | --- | --- | --- | --- | --- | --- | --- | --- | --- |
| 16S rDNA |  | y = 18.984x + 0.0243 R² = 0.6308 | y = 5.5533x + 0.0109 R² = 0.6333 | y = 28.544x + 0.0294 R² = 0.6753 | y = 19.403x + 0.0309 R² = 0.4775 | y = 20.877x + 0.0282 R² = 0.6188 | y = 29.927x + 0.0323 R² = 0.7105 | y = 24.285x + 0.0292 R² = 0.6358 | y = 20.332x + 0.0258 R² = 0.6643 |
| *gyrB* | y = 0.0332x - 0.0002 R² = 0.6308 |  | y = 0.2551x + 0.006 R² = 0.7633 | y = 1.274x + 0.0061 R² = 0.7687 | y = 0.9365x + 0.011 R² = 0.6355 | y = 0.9373x + 0.0108 R² = 0.7126 | y = 1.3872x + 0.0049 R² = 0.8722 | y = 1.1457x + 0.0058 R² = 0.8084 | y = 0.9522x + 0.0066 R² = 0.8324 |
| *rpoB* | y = 0.114x - 0.0006 R² = 0.6333 | y = 2.9925x - 0.0041 R² = 0.7633 |  | y = 4.6934x - 0.0175 R² = 0.8891 | y = 3.3222x - 0.0037 R² = 0.6817 | y = 3.5691x - 0.0089 R² = 0.8807 | y = 4.7558x - 0.0134 R² = 0.8737 | y = 4.0634x - 0.0121 R² = 0.8667 | y = 3.3771x - 0.0083 R² = 0.8924 |
| *aroE* | y = 0.0237x - 0.0001 R² = 0.6753 | y = 0.6033x + 0.0097 R² = 0.7687 | y = 0.1894x + 0.0056 R² = 0.8891 |  | y = 0.7304x + 0.0068 R² = 0.8163 | y = 0.7546x + 0.0048 R² = 0.9754 | y = 0.9926x + 0.0059 R² = 0.943 | y = 0.8595x + 0.0035 R² = 0.9606 | y = 0.7116x + 0.0049 R² = 0.9817 |
| *mutL* | y = 0.0246x + 0.0002 R² = 0.4775 | y = 0.6786x + 0.0137 R² = 0.6355 | y = 0.2052x + 0.0074 R² = 0.6817 | y = 1.1176x + 0.0071 R² = 0.8163 |  | y = 0.854x + 0.0094 R² = 0.8165 | y = 1.1171x + 0.0124 R² = 0.7806 | y = 0.9869x + 0.0078 R² = 0.8278 | y = 0.8283x + 0.0078 R² = 0.8693 |
| *pycA* | y = 0.0296x - 0.0002 R² = 0.6188 | y = 0.7603x + 0.0084 R² = 0.7126 | y = 0.2467x + 0.0047 R² = 0.8807 | y = 1.2925x - 0.0042 R² = 0.9754 | y = 0.956x + 0.003 R² = 0.8165 |  | y = 1.272x + 0.0024 R² = 0.904 | y = 1.1122x - 0.0002 R² = 0.9392 | y = 0.9226x + 0.0017 R² = 0.9634 |
| *pyrE* | y = 0.0237x - 0.0003 R² = 0.7105 | y = 0.6287x + 0.0043 R² = 0.8722 | y = 0.1837x + 0.0051 R² = 0.8737 | y = 0.95x - 0.0011 R² = 0.943 | y = 0.6987x + 0.0057 R² = 0.7806 | y = 0.7107x + 0.0045 R² = 0.904 |  | y = 0.831x + 0.0013 R² = 0.9384 | y = 0.6905x + 0.003 R² = 0.9658 |
| *trpB* | y = 0.0262x - 0.0001 R² = 0.6358 | y = 0.7056x + 0.007 R² = 0.8084 | y = 0.2133x + 0.0054 R² = 0.8667 | y = 1.1177x - 0.0007 R² = 0.9606 | y = 0.8388x + 0.0047 R² = 0.8278 | y = 0.8445x + 0.0042 R² = 0.9392 | y = 1.1292x + 0.0037 R² = 0.9384 |  | y = 0.8107x + 0.0033 R² = 0.9797 |
| 7 concatenated genes | y = 0.0327x - 0.0002 R² = 0.6643 | y = 0.8742x + 0.0039 R² = 0.8324 | y = 0.2643x + 0.0044 R² = 0.8924 | y = 1.3796x - 0.0053 R² = 0.9817 | y = 1.0495x + 0.0003 R² = 0.8693 | y = 1.0442x + 0.0006 R² = 0.9634 | y = 1.3987x - 0.0012 R² = 0.9658 | y = 1.2085x - 0.0025 R² = 0.9797 |  |

**Table S6 The characteristics comparison of the single gene and concatenated genes in three species**

| Species | Characteristics | Locus (Length bp) | | | | | | | |
| --- | --- | --- | --- | --- | --- | --- | --- | --- | --- |
| (Strain number) |  | *gyrB* (717bp) | *rpoB* (927bp) | *aroE* (900bp) | *mutL* (828bp) | *pycA* (864bp) | *pyrE* (546bp) | *trpB* (867bp) | Seven concatenated genes (5649bp) |
| *B. altitudinis* (49) | No.of Alleles | 22 | 21 | 16 | 22 | 23 | 19 | 15 | 37 |
|  | Polymorphic sites number (%) | 71 (9.902) | 39 (4.207) | 41 (4.556) | 43 (5.193) | 59 (6.829) | 27 (4.945) | 32 (3.691) | 313 (5.541) |
|  | Mean G+C content (mol%) | 42.3 | 45.4 | 42.6 | 45.2 | 42.6 | 45.8 | 44.5 | 44.0 |
|  | K_2_P distance  range (mean) | 0.000-0.061 (0.011) | 0.000-0.023 (0.007) | 0.000-0.022 (0.009) | 0.000-0.021 (0.010) | 0.000-0.032 (0.013) | 0.000-0.026 (0.012) | 0.000-0.020 (0.008) | 0.000-0.021 (0.010) |
| *B. pumilus* (13) | No.of Alleles | 4 | 5 | 5 | 7 | 4 | 4 | 4 | 7 |
|  | Polymorphic sites number (%) | 24 (3.347) | 18 (1.942) | 48 (5.333) | 30 (3.623) | 36 (4.167) | 28 (5.128) | 45 (5.190) | 229 (4.054) |
|  | Mean G+C content (mol%) | 41.1 | 46.3 | 41.5 | 46.4 | 44.3 | 47.5 | 44.8 | 44.5 |
|  | K_2_P distance  range (mean) | 0.000-0.032 (0.007) | 0.000-0.018 (0.006) | 0.000-0.050 (0.015) | 0.000-0.031 (0.010) | 0.000-0.040 (0.012) | 0.000-0.048 (0.014) | 0.000-0.050 (0.015) | 0.000-0.037 (0.011) |
| *B. safensis* (13) | No.of Alleles | 7 | 6 | 7 | 6 | 7 | 7 | 7 | 10 |
|  | Polymorphic sites number (%) | 44 (6.137) | 24 (2.589) | 55 (6.111) | 56 (6.763) | 33 (3.819) | 42 (7.692) | 61 (7.036) | 382 (6.762) |
|  | Mean G+C content (mol%) | 42.6 | 46.3 | 41.5 | 45.2 | 42.6 | 47.6 | 45.6 | 44.3 |
|  | K_2_P distance  range (mean) | 0.000-0.040 (0.024) | 0.000-0.019  (0.008) | 0.000-0.041  (0.018) | 0.000-0.053  (0.029) | 0.000-0.024 (0.012) | 0.000-0.048 (0.030) | 0.000-0.043 (0.022) | 0.000-0.040 (0.023) |

**Table S7 The number of strain pairs within different similarity grades of the housekeeping genes of 79 strains**

|  | The number of strain pairs | | | | | | | |
| --- | --- | --- | --- | --- | --- | --- | --- | --- |
| Range of similarities | *gyrB* | *rpoB* | *pycA* | *pyrE* | *mutL* | *aroE* | *trpB* | Seven concatenated genes |
| 82.00-82.99 | 0 | 0 | 0 | 159 | 0 | 0 | 0 | 0 |
| 83.00-83.99 | 0 | 0 | 0 | 171 | 0 | 0 | 0 | 0 |
| 84.00-84.99 | 0 | 0 | 0 | 292 | 0 | 70 | 0 | 0 |
| 85.00-85.99 | 0 | 0 | 0 | 117 | 0 | 897 | 97 | 0 |
| 86.00-86.99 | 0 | 0 | 0 | 547 | 43 | 380 | 38 | 0 |
| 87.00-87.99 | 0 | 0 | 578 | 204 | 480 | 21 | 1132 | 0 |
| 88.00-88.99 | 2 | 0 | 247 | 68 | 640 | 84 | 169 | 189 |
| 89.00-89.99 | 507 | 0 | 501 | 113 | 249 | 37 | 150 | 1159 |
| 90.00-90.99 | 441 | 0 | 30 | 53 | 152 | 59 | 4 | 80 |
| 91.00-91.99 | 140 | 0 | 163 | 24 | 169 | 135 | 135 | 106 |
| 92.00-92.99 | 33 | 0 | 73 | **0** | 12 | 65 | 10 | 201 |
| 93.00-93.99 | 583 | 0 | 122 | **0** | 3 | **0** | 7 | 3 |
| 94.00-94.99 | 36 | 0 | 31 | **0** | 26 | **0** | 6 | 10 |
| 95.00-95.99 | 6 | 116 | 6 | 59 | 10 | 23 | 25 | **0** |
| 96.00-96.99 | 55 | 678 | 35 | 17 | 1 | 13 | 33 | 46 |
| 97.00-97.99 | 66 | 800 | 231 | 159 | 23 | 36 | 11 | 12 |
| 98.00-98.99 | 348 | 582 | 497 | 637 | 764 | 579 | 608 | 628 |
| 99.00-99.99 | 615 | 622 | 319 | 274 | 321 | 320 | 249 | 557 |
| 100 | 249 | 283 | 248 | 187 | 188 | 362 | 407 | 90 |

Zero in red means interspecies gap

**Table S8 The Ka/Ks ratio of 7 single housekeeping genes in different species and all the tested strains of the *B. pumilus* group**

| Bacteria | *gyrB* | *rpoB* | *aroE* | *pycA* | *pyrE* | *mutL* | *trpB* |
| --- | --- | --- | --- | --- | --- | --- | --- |
| *B. altitudinis* | 0.0156 | 0.0068 | 0.0383 | 0.0252 | 0.1200 | 0.0304 | 0.0064 |
| *B. pumilus* | 0.0352 | 0.0000 | 0.0800 | 0.0000 | 0.0134 | 0.0310 | 0.0199 |
| *B. safensis* | 0.0296 | 0.0098 | 0.0561 | 0.0044 | 0.0288 | 0.0000 | 0.0144 |
| All strains | 0.0265 | 0.0034 | 0.0473 | 0.0188 | 0.0731 | 0.0199 | 0.0198 |

The values in red: the highest in each species group.
